# The genetics of resilience and its relationships with egg production traits and antibody traits in chickens

**DOI:** 10.1186/s12711-024-00888-5

**Published:** 2024-03-19

**Authors:** Tom V. L. Berghof, Nicolas Bedere, Katrijn Peeters, Marieke Poppe, Jeroen Visscher, Han A. Mulder

**Affiliations:** 1 Wageningen University & Research Animal Breeding and Genomics, PO Box 338, 6700 AH Wageningen, The Netherlands; 2 Reproductive Biotechnology, TUM School of Life Sciences, Technical University of Munich, Liesel-Beckmann-Strasse 1, 85354 Freising, Germany; 3 PEGASE, INRAE, Institut Agro, 35590 Saint Gilles, France; 4 Hendrix Genetics B.V., P.O. Box 114, 5830 AC Boxmeer, The Netherlands; 5 CRV B.V., Wassenaarweg 20, Arnhem, The Netherlands

## Abstract

**Background:**

Resilience is the capacity of an animal to be minimally affected by disturbances or to rapidly return to its initial state before exposure to a disturbance. Resilient livestock are desired because of their improved health and increased economic profit. Genetic improvement of resilience may also lead to trade-offs with production traits. Recently, resilience indicators based on longitudinal data have been suggested, but they need further evaluation to determine whether they are indeed predictive of improved resilience, such as disease resilience. This study investigated different resilience indicators based on deviations between expected and observed egg production (EP) by exploring their genetic parameters, their possible trade-offs with production traits, and their relationships with antibody traits in chickens.

**Methods:**

Egg production in a nucleus breeding herd environment based on 1-week-, 2-week-, or 3-week-intervals of two purebred chicken lines, a white egg-laying (33,825 chickens) and a brown egg-laying line (34,397 chickens), were used to determine deviations between observed EP and expected average batch EP, and between observed EP and expected individual EP. These deviations were used to calculate three types of resilience indicators for two life periods of each individual: natural logarithm-transformed variance (ln(variance)), skewness, and lag-one autocorrelation (autocorrelation) of deviations from 25 to 83 weeks of age and from 83 weeks of age to end of life. Then, we estimated their genetic correlations with EP traits and with two antibody traits.

**Results:**

The most promising resilience indicators were those based on 1-week-intervals, as they had the highest heritability estimates (0.02–0.12) and high genetic correlations (above 0.60) with the same resilience indicators based on longer intervals. The three types of resilience indicators differed genetically from each other, which indicates that they possibly capture different aspects of resilience. Genetic correlations of the resilience indicator traits based on 1-week-intervals with EP traits were favorable or zero, which means that trade-off effects were marginal. The resilience indicator traits based on 1-week-intervals also showed no genetic correlations with the antibody traits, which suggests that they are not informative for improved immunity or vice versa in the nucleus environment.

**Conclusions:**

This paper gives direction towards the evaluation and implementation of resilience indicators, i.e. to further investigate resilience indicator traits based on 1-week-intervals, in breeding programs for selecting genetically more resilient layer chickens.

**Supplementary Information:**

The online version contains supplementary material available at 10.1186/s12711-024-00888-5.

## Background

Resilience is the capacity of an animal to be minimally affected by disturbances or to rapidly return to its initial state before exposure to a disturbance [1, 2]. It has been shown that resilience has an economic value since labor costs, health costs, and production losses are lower for more resilient livestock [2, 3]. However, the ‘best’ resilience indicator or combination of resilience indicators to be included in a selection index is still under investigation.

One approach to obtaining resilience indicators is based on deviations between observed and expected production [1, 4–6]. Such deviations for production traits and their patterns over time have been shown to be phenotypically indicative of health or health-related traits (e.g. [7–15]). These deviations, also known as uniformity of traits, also have a genetic component, e.g. residual variation of eggshell color [16], egg weight [17], and body weight [18–20] were found to be heritable in chickens. In the last decade, this approach has received more attention from the quantitative genetics field due to (expected) technological developments that allow the collection of longitudinal data, and to the acquired capability and expertise to handle and analyze ‘big data’ for genetic evaluation by using the deviations in these data for an individual [21]. Based on this, Berghof et al. [2] proposed three resilience indicators that consider disturbances that differ in nature during a production cycle: natural logarithm-transformed variance (ln(variance)) of deviations, skewness of deviations, and autocorrelation of deviations between observed and expected production [2]. More resilient animals are expected to have a more uniform production with fewer and smaller deviations compared to less resilient animals, because they are less influenced by disturbances. Thus, they are expected to have a small ln(variance), and a skewness and an autocorrelation around zero [2].

Until now, a limited number of studies have estimated the genetic parameters of these resilience indicators. However, in dairy cattle, the ln(variance), skewness, and autocorrelation of the deviations of daily milk production [22–25] and of fluctuations in step count [26] were estimated to be heritable. In layer chickens, these three resilience indicators based on deviations of 4-weekly body weight measurements and of egg production (EP) were also found to have a genetic component [27, 28]. However, except for Poppe et al. [23], who compared different models to estimate lactation curves, the effects of different time intervals or production estimates on resilience indicator estimates have thus far been ignored. This is likely in part due to the lack of high-resolution longitudinal phenotypes recorded in livestock species. A more detailed understanding of the impact of observation frequencies and of different methods to estimate deviations is important to further define and understand resilience indicators for livestock breeding.

In addition, the proposed resilience indicators lack a clear physiological understanding. On the one hand, improved resilience could lead to decreased production as a result of energy relocation (trade-off). This was for example observed for resilience indicators based on daily milk production and milk yield [23, 29], although the economic value of a more resilient animal with lower production might still be higher over a lifetime than that of a low resilient animal with higher production (see Poppe et al. [30]). On the other hand, improved resilience should lead to, among others, improved immunity and disease resistance. Indeed, the variance of the deviations for finishing pigs of daily feed intake and daily duration at the feeder were favorably genetically correlated with mortality and number of treatments in a ‘natural disease challenge environment’ [31]. The ln(variance) of the deviations of daily milk production in dairy cows was also favorably genetically correlated with a lower incidence of milk production-related diseases and greater longevity [22–24]. Similarly, the autocorrelation of deviations and the mean of negative residuals of step count showed potential as resilience indicators in dairy cattle [26]. However, in chickens, ln(variance) was found to have only a weak relationship with resistance to avian pathogenic *Escherichia coli* (APEC), and no genetic correlation with another known disease resistance indicator (i.e. titer of natural antibodies (NAb)) [27]. Thus, more work is needed to understand the biology that underlies these resilience indicators and to confirm their relationships with known physiological systems, such as the immune system.

In this study, we used longitudinally collected observations on a large number of individuals from two purebred chicken lines to study the possibility and feasibility to genetically improve resilience in chickens. We estimated the heritability of different resilience indicators based on deviations of EP, as well as their genetic correlations with production and immune traits. Thus, the objectives of this study were to investigate:

1. the effects of different EP intervals (i.e. 1-week, 2-week, and 3-week) and different expected EP references (i.e. average batch EP and individual EP) on genetic parameters of three resilience indicator types (i.e. ln(variance), skewness, and autocorrelation) for two life periods (i.e. from 25 to 83 weeks of age and after 83 weeks of age);
2. potential trade-offs between the resilience indicators and EP by estimating the genetic correlations between selected resilience indicators and EP traits;
3. the predictive potential of the resilience indicators for immunity traits and vice versa by estimating genetic correlations between selected resilience indicators and keyhole limpet hemocyanin (KLH)-binding IgM and IgG NAb titers.

## Methods

### Animal populations

Two purebred Leghorn chicken lines from Hendrix Genetics were used for this study: one white egg-laying line, which in some other works is referred to as ‘WA’ and here as ‘White’, and one brown egg-laying line, which in some other works is referred to as ‘BD’ and here as ‘Brown’. Data were collected between 2012 and 2018 on 39 batches at 13 breeding nucleus locations for White and on 41 batches for Brown at 13 breeding nucleus locations. Generally, one breeding nucleus location (‘location’ in the statistical model) contributed three batches (‘batch’ in the statistical model), typically consecutive hatches with a two-week-interval. Chickens were housed in individual cages, which were organized in rows and levels, for which a unique identifier was created (‘row*level’ in the statistical model). Birds were kept according to standard Hendrix Genetics protocols.

It should be noted that the populations studied here, part of the phenotypes, and part of the results were previously described in Bedere et al. [28], Doekes et al. [32], and Berghof et al. [33, 34]. Bedere et al. [28] investigated the genetic characteristics of the purebred and crossbred individuals and the purebred-crossbred correlations of the resilience indicators based on average batch production for the laying period from 25 to 83 weeks of age with 1-week-intervals. Doekes et al. [32] investigated the genetic characteristics of the NAb titers and the resilience indicators for Brown. The studies of Berghof et al. [33, 34] investigated the genetic characteristics of the NAb titers for White. Our study is complementary to these previous studies, because it focuses on the genetic characteristics of the resilience indicators based on different definitions of expected production (i.e. different interval lengths, different life periods, and expected batch vs. individual production), and their genetic correlations with the EP and antibody traits.

### Phenotypes

**Fig. 1:**
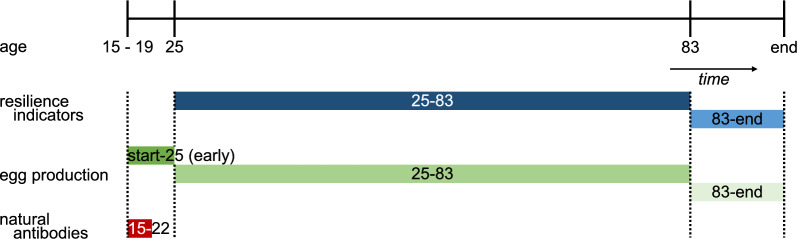
Schematic overview of individual trait measurements: resilience indicators, egg production, and natural antibody levels. Top bar shows the age of the individuals in weeks with relevant time points during the production cycle. Individual caging after rearing takes place between 15 and 19 weeks of age. Traditionally, breeding programs of laying hens have focused on egg production between 25 and 83 weeks of age, but currently, they focus on prolonging the egg production cycle (to more than 100 weeks). Therefore, the relevant periods in this study are split up into: the full production cycle (i.e. all eggs from start to end), the early production cycle (i.e. from start to 25 weeks of age), the 'traditional' production cycle (i.e. 25 to 83 weeks of age), and the time period after the 'traditional' production cycle (i.e. 83 weeks of age to end)

An overview of the collection time for each phenotype is in Fig. 1 and the descriptive statistics for all phenotypes are in Table 1. Note that the resilience indicators and EP traits could only be recorded on females, while the antibody traits were recorded on both sexes.

**Table 1:** Descriptive statistics^a^ for each trait

Trait		Period (weeks of age)	Interval	White	Brown
Average batch production	ln(variance)	25 to 83	1 week	− 0.81 (0.94) *31,925*	− 0.33 (1.09) *1462*
			2 weeks	0.06 (1.01) *31,812*	0.66 (1.23) *31,381*
			3 weeks	0.50 (1.07) *31,707*	1.21 (1.30) *31,279*
		83 to end	1 week	− 0.33 (1.10) *29,979*	− 0.26 (1.26) *26,760*
			2 weeks	0.47 (1.22) *29,660*	0.65 (1.39) *19,317*
			3 weeks	0.85 (1.35) *25,196*	1.42 (1.36) *2934*
	Skewness	25 to 83	1 week	− 1.50 (1.04) *31,919*	− 1.19 (1.15) *31,502*
			2 weeks	− 0.97 (1.02) *31,867*	− 0.79 (1.11) *31,415*
			3 weeks	− 0.66 (0.91) *31,751*	− 0.55 (1.02) *31,304*
		83 to end	1 week	− 0.63 (0.71) *29,952*	− 0.32 (0.69) *26,747*
			2 weeks	− 0.29 (0.56) *29,662*	− 0.16 (0.51) *19,317*
			3 weeks	− 0.14 (0.46) *25,240*	− 0.09 (0.46) *2951*
	Autocorrelation	25 to 83	1 week	0.21 (0.24) *31,994*	0.36 (0.29) *31,524*
			2 weeks	0.14 (0.29) *31,877*	0.36 (0.29) *31,414*
			3 weeks	0.11 (0.30) *31,761*	0.33 (0.30) *31,304*
		83 to end	1 week	0.12 (0.31) *29,992*	0.13 (0.35) *26,760*
			2 weeks	0.01 (0.32) *29,662*	− 0.01 (0.34) *19,317*
			3 weeks	− 0.09 (0.31) *25,240*	− 0.01 (0.32) *2951*
Individual production	ln(variance)	25 to 83	1 week	− 0.61 (1.09) *31,816*	− 0.63 (1.12) *31,381*
		83 to end	1 week	− 0.36 (1.06) *29,693*	− 0.26 (1.33) *26,478*
	Skewness	25 to 83	1 week	− 2.59 (1.12) *31,782*	− 2.01 (1.19) *31,264*
		83 to end	1 week	− 0.84 (0.76) *29,711*	− 0.46 (0.70) *26,594*
	Autocorrelation	25 to 83	1 week	0.23 (0.21) *31,839*	0.22 (0.28) *31,457*
		83 to end	1 week	0.07 (0.29) *29,723*	0.10 (0.34) *26,641*
Egg production (number)		Full		486.0 (84.7) *32,056*	427.4 (95.9) *31,603*
		Early		29.2 (8.7) *32,056*	37.4 (7.9) *31,603*
		25 to 83		369.3 (57.1) *32,056*	345.8 (73.0) *31,603*
		83 to end		92.5 (31.7) *30,339*	51.3 (26.0) *27,159*
Natural antibodies (titers)	IgM	Once between 15 and 22		7.10 (1.43) *4856*^b^	6.74 (1.20) *2789*^c^
	IgG	Once between 15 and 22		6.16 (1.57) *4852*^d^	6.02 (1.29) *2789*^c^

^a^Average, standard deviation (in parentheses), and number of observations used (in *italics*) for each trait: resilience indicators based on average batch production, resilience indicators based on expected individual production, egg production for each life period, and keyhole limpet hemocyanin (KLH)-binding natural antibody for White and Brown

^b^Consisting of 3100 females and 1756 males

^c^Consisting of 2509 females and 280 males

^d^Consisting of 3096 females and 1756 males

#### Resilience indicators

After rearing, individual housing started between 15 and 19 weeks of age (‘start’). Egg production was recorded from the start to the end of a female’s life (‘end’), which was either (1) at the end of the production life of a batch (i.e. when the stable was emptied), which was generally between 91 and 105 weeks of age, (2) when the bird was found with severe injuries and consequently euthanized, or (3) when the bird was found dead by animal care takers.

Resilience indicators were assessed from 25 to 83 weeks of age (25–83) and from 83 weeks of age to end (83-end). Egg production from the start of individual caging to 25 weeks of age (early EP; start-25) was not used for estimating the resilience indicators, because it is considered to be a genetically different trait from EP during later stages of life. Indeed EP in the start-25 period is strongly influenced by the sexual maturity of the individual, so variation in EP may be more representative of the stage and speed of sexual development than of resilience (J. Visscher, personal communication). The resilience indicators were also estimated from 25 weeks of age to end, but are not reported, because they were found to be highly genetically correlated with the corresponding resilience indicators for the 25–83 period and can, therefore, be considered very similar. The division of the EP period until or from 83 weeks of age was based on the traditional design of breeding programs, which are strongly focused on EP from 25 to 83 weeks of age (EP 25–83). Thus, it can be expected that variation in EP is minimal due to the strong selection for EP during this period, which has been practiced for many generations. However, since modern breeding programs put more emphasis on prolonging the EP cycle (from 83 to more than 100 weeks of age), greater variation in EP from 83 weeks of age to end (EP 83-end) may still exist. Thus, to investigate these possible differences, resilience indicators were based on the 25–83 and 83-end life periods.

Egg production was generally recorded on a one-day- to four-day-interval throughout the production cycle (i.e. from 25 weeks of age to end). However, EP was registered over longer intervals for some individuals within a batch for no obvious reason. Similarly, EP for some collection intervals was not registered for some birds, for no obvious reason, and in these cases, missing EP was estimated by Hendrix Genetics staff based on EP before and after this collection interval. Nevertheless, because these data were used for longer intervals and the estimated data were included in the database and are used in the breeding programs of Hendrix Genetics, they were also included in our data in order to make our study more applicable for practical breeding purposes.

Average daily EP per female was calculated based on the observed production over a certain time interval. Although birds only lay one egg per day, it is possible to have an observation of two eggs per day when the first egg was laid after egg collection on day 1 and the second egg was laid before egg collection on day 2. However, females with an average daily EP observation of more than two eggs were removed from the datasets because this is physiologically not possible.

For all resilience indicators, EP deviations were calculated for each individual as the difference between an individual’s observed and expected EP during non-overlapping 1-week-, 2-week-, or 3-week-intervals for the two 25–83 and 83-end periods (see ‘Discussion’ for the rationale behind this). The expected EP was set to the average batch EP, similar to the studies of Berghof et al. [27] and Bedere et al. [28]. For example, an individual’s EP in the 30th week was compared to its batch’s average in the 30th week. However, other studies have investigated resilience indicators using the deviations from expected production derived using individual production (e.g. [22–25, 31]). Because the data used here allowed for a comparison of both methods the expected individual production was estimated in a similar manner as in Poppe et al. [23–26] for a selected set of resilience indicators (see ‘Results’) based on the individual’s EP production curve. The latter was estimated separately for each individual by fitting a 4th order 0.7 quantile polynomial regression to 1-week-interval EP observations from 25 weeks of age to end. By using a 0.7 quantile regression, the estimated curve was less sensitive to drops in EP, i.e. low EP values had less influence on the predicted EP curve than high EP values [26]. For more information on the rationale for using quantile regression, see Poppe et al. [26]. The expected EP curve was estimated using R and the R-packages ‘quantreg’ and ‘dplyr’ [35–37].

The following resilience indicators were computed for each individual: the natural logarithm (ln)-transformed variance of deviations (ln(variance)), the skewness of deviations (skewness), and the lag-one autocorrelation of deviations (autocorrelation), as proposed by Berghof et al. [2]. The variance was ln-transformed, because variance phenotypes typically display a right-skewed distribution, which is removed by the ln-transformation. In addition, ln is the commonly used scale to express variance phenotypes (also known as uniformity) in other studies and, therefore, allows direct comparison of genetic parameter estimates between studies [38, 39].

The resilience indicators were calculated for individuals with five or more interval observations for the specific period. This resulted in fewer, but still a large number of observations for the resilience indicators based on the 83-end period (n ≥ 29,317), except for the resilience indicators for this period computed based on 3-week-intervals for Brown (n = 2934–2951) (see Table 1). Resilience indicators were calculated using R and the R-packages ‘dplyr’ and ‘e1071’ [35, 37, 40]. Individual estimates for a resilience indicator were deleted from the dataset when they deviated by more than four standard deviations from the average of the whole population.

In total, 24 resilience indicators were investigated, of which 18 were based on deviations from average batch production and six on deviations from expected individual production. Resilience indicators based on average batch production included the three resilience indicators (ln(variance), skewness, and autocorrelation) for two periods (25–83 and 83-end) and based on three intervals (1-week, 2-week, or 3-week). Resilience indicators based on individual production were limited to the three resilience indicators (ln(variance), skewness, and autocorrelation) for two periods (25–83 and 83-end) and based the 1-week interval, based on the average batch production results (see ‘Results’).

#### Egg production traits

Individual EP was evaluated for four periods: start–end (i.e. full production cycle), start-25 (early EP), 25–83, and 83-end. The choice of these periods was based on a similar rationale as described for the choice of periods for the resilience indicators.

#### Antibody traits

Two NAb isotypes were analyzed as antibody traits: IgM and IgG (also named IgY in birds). Plasma samples were collected on a subset of birds between 15 and 22 weeks of age for White and between 16 and 20 weeks of age for Brown. The Brown population did not finish its production cycle at the moment resilience indicators were determined, thus relationships between NAb and resilience indicators can only be determined through the relationship matrix. NAb optical densities (OD) were determined in individual plasma samples by an indirect two-step ELISA as described by Berghof et al. [34]. Antibody titers were calculated as described by Berghof et al. [34], based on Frankena [41].

### Statistical analyses

Individuals were divided into classes based on their survival (‘maximum age-class’) to account for the number of observations that contributed to the resilience indicators. Maximum age-classes were defined based on mortality before 30 weeks of age (i.e. between 25 and 30 weeks of age) and on 4-week-intervals thereafter (i.e. mortality between 30 and 34 weeks of age, between 34 and 38 weeks of age, etc.).

Bivariate analyses were performed to investigate relationships between resilience indicators and EP traits or antibody traits, following the definitions of statistical models for EP and antibody traits based on univariate analyses. Although not part of the aim of this paper, the results of these analyses are reported in Additional file 1: Table S8 for the EP traits and Additional file 1: Table S13 for the antibody traits. Phenotypic correlations for all the genetic correlations reported here were also estimated and are reported in Additional file 1: Tables S1 to S7, Additional file 1: Tables S9 to S12, and Additional file 1: Tables S14 to S16, but will not be further discussed. Maternal genetic effects were assessed but found not to be significant (results not reported), while maternal environmental effects were significant for several traits and are reported in the ‘Results’ section.

All statistical analyses were conducted separately for White and Brown. Fixed effects in the statistical models were location, batch, row*level, and maximum age-class. Statistical analyses were performed using ASReml 4.1 [42] until convergence or for a maximum of 50 iterations. Significance was declared for p-values lower or equal to 0.05, and a tendency to significance was declared for p-values lower or equal to 0.10.

The linear animal model for estimating the variance components of the resilience indicators was:

1 $${\mathbf{y}}_{\mathbf{i}\mathbf{j}\mathbf{k}\mathbf{l}\mathbf{m}}=\upmu +{\mathbf{b}\mathbf{a}\mathbf{t}\mathbf{c}\mathbf{h}}_{\mathbf{i}}+\left({\mathbf{l}\mathbf{o}\mathbf{c}}_{\mathbf{j}}*{\mathbf{r}\mathbf{l}}_{\mathbf{k}}\right)+{\mathbf{m}\mathbf{a}\mathbf{c}}_{\mathbf{l}}+{\mathbf{a}}_{\mathbf{m}}+{\mathbf{e}}_{\mathbf{i}\mathbf{j}\mathbf{k}\mathbf{l}\mathbf{m}},$$

where ${\mathbf{y}}_{\mathbf{i}\mathbf{j}\mathbf{k}\mathbf{l}\mathbf{m}}$ is the vector of any of the 24 resilience indicators; $\upmu$ is the overall mean, ${\mathbf{b}\mathbf{a}\mathbf{t}\mathbf{c}\mathbf{h}}_{\mathbf{i}}$ is the vector of the fixed effect of batch (${\text{i}}$ = 1 to 39 for White and ${\text{i}}$ = 1 to 41 for Brown), $\left({\mathbf{l}\mathbf{o}\mathbf{c}}_{\mathbf{j}}\mathbf{*}{\mathbf{r}\mathbf{l}}_{\mathbf{k}}\right)$ is the vector of the fixed effect of the position of an individual’s cage at a location ${\text{j}}$ with ${\mathbf{l}\mathbf{o}\mathbf{c}}_{\mathbf{j}}$ being the vector of the fixed effect of location (${\text{j}}$ = 1 to 13 for White and ${\text{j}}$ = 1 to 13 for Brown) and ${\mathbf{r}\mathbf{l}}_{\mathbf{k}}$ being the vector of the fixed effect of the row*level-identifier (${\text{k}}$ = 1 to 35 for White and ${\text{k}}$ = 1 to 49 for Brown), ${\mathbf{m}\mathbf{a}\mathbf{c}}_{\mathbf{l}}$ is the vector of the fixed effect of maximum age-class (${\text{l}}$ = 1 to 20 for White and ${\text{l}}$ = 1 to 19 for Brown), ${\mathbf{a}}_{\mathbf{m}}$ is the vector of the random additive genetic effect of the ${\text{m}}$^th^ individual, assumed to follow ~ ${\text{N}}(0, \mathbf{A}{\upsigma }_{{\text{a}}}^{2}$), and ${\mathbf{e}}_{\mathbf{i}\mathbf{j}\mathbf{k}\mathbf{l}\mathbf{m}}$ is the vector of residuals, assumed to follow $\sim {\text{N}}(0, \mathbf{I}{\upsigma }_{{\text{e}}}^{2}$). Assumed (co)variance structures for the random model terms are $\mathbf{A}{\upsigma }_{{\text{a}}}^{2}$ and $\mathbf{I}{\upsigma }_{{\text{e}}}^{2}$, where $\mathbf{A}$ is the additive genetic relationship matrix based on the pedigree consisting of 497,541 individuals across 23 generations for White and 325,811 individuals across 18 generations for Brown, ${\upsigma }_{{\text{a}}}^{2}$ is the additive genetic variance, $\mathbf{I}$ is an identity matrix, and ${\upsigma }_{{\text{e}}}^{2}$ is the residual variance.

Model (1) was extended by adding a maternal environmental effect to test for its significance:

2 $${\mathbf{y}}_{\mathbf{i}\mathbf{j}\mathbf{k}\mathbf{l}\mathbf{m}\mathbf{n}}=\upmu +{\mathbf{b}\mathbf{a}\mathbf{t}\mathbf{c}\mathbf{h}}_{\mathbf{i}}+\left({\mathbf{l}\mathbf{o}\mathbf{c}}_{\mathbf{j}}\mathbf{*}{\mathbf{r}\mathbf{l}}_{\mathbf{k}}\right)+{\mathbf{m}\mathbf{a}\mathbf{c}}_{\mathbf{l}}+{\mathbf{a}}_{\mathbf{m}}+{\mathbf{d}\mathbf{a}\mathbf{m}}_{\mathbf{n}}+{\mathbf{e}}_{\mathbf{i}\mathbf{j}\mathbf{k}\mathbf{l}\mathbf{m}\mathbf{n},}$$

with all components as defined for Model (1) and ${\mathbf{d}\mathbf{a}\mathbf{m}}_{\mathbf{n}}$ is the vector of the random environmental effect of the ${\text{n}}$^th^ dam, assumed to follow $\sim {\text{N}}(0, \mathbf{I}{\upsigma }_{{\text{m}}}^{2}$), where $\mathbf{I}$ is an identity matrix and ${\upsigma }_{{\text{m}}}^{2}$ the maternal environmental variance. Significance was assessed with a likelihood ratio test (LRT) assuming that the likelihood ratio follows a ${\upchi }_{1}^{2}$-distribution, comparing nested models with or without the random effect tested. Resilience indicators for which ${\upsigma }_{{\text{a}}}^{2}$ were not significantly different from zero, and thus were not further investigated.

The linear animal model used to estimate the variance components of the EP traits was the same as Model (1) or Model (2) when the maternal environmental effect was significant.

The linear animal model for estimating the variance components of the NAb titers was:

3 $${\mathbf{y}}_{\mathbf{i}\mathbf{j}\mathbf{k}\mathbf{l}}=\upmu +{\mathbf{p}\mathbf{l}\mathbf{a}\mathbf{t}\mathbf{e}}_{\mathbf{i}}+{\upbeta }_{1}*{{\text{age}}}_{{\text{j}}}+{\mathbf{s}\mathbf{e}\mathbf{x}}_{\mathbf{k}}+{\mathbf{a}}_{\mathbf{l}}+{\mathbf{e}}_{\mathbf{i}\mathbf{j}\mathbf{k}\mathbf{l}},$$

where ${\mathbf{y}}_{\mathbf{i}\mathbf{j}\mathbf{k}\mathbf{l}}$ is the vector of the titer of KLH-binding IgM or IgG NAb titer, $\upmu$ is the overall mean, ${\mathbf{p}\mathbf{l}\mathbf{a}\mathbf{t}\mathbf{e}}_{\mathbf{i}}$ is the vector of the fixed effect of the plate ${\text{i}}$ on which a sample was analyzed (${\text{i}}$ = 1–257), ${{\text{age}}}_{{\text{j}}}$ is the covariate describing the effect of age at sampling (${\text{j}}$ = 15–22) with regression coefficient ${\upbeta }_{1}$, ${\mathbf{s}\mathbf{e}\mathbf{x}}_{\mathbf{k}}$ is the vector of the fixed effect of sex (${\text{k}}$ = male or female), ${\mathbf{a}}_{\mathbf{l}}$ is the vector of the random additive genetic effect of the ${\text{l}}$^th^ individual assumed to follow $\sim {\text{N}}(0, \mathbf{A}{\upsigma }_{{\text{a}}}^{2}$), ${\mathbf{e}}_{\mathbf{i}\mathbf{j}\mathbf{k}\mathbf{l}}$ is the vector of the residual term, assumed to follow $\sim {\text{N}}(0, \mathbf{I}{\upsigma }_{{\text{e}}}^{2}$). The assumed (co)variance structures of the random model terms are equal to the (co)variance structures of the random model terms of the resilience indicators. For analyses of White, the effect of sex was removed from the model because the plate effect accounts for the confounded effects on the samples, including sex, storage, and effects of the analysis [33, 34]. Samples for Brown were randomized over the used plates; thus, the sex effect can be accounted for separately in the model.

Based on estimates of variance components from the respective univariate models, estimates of heritabilities (${{\text{h}}}^{2}$) were calculated as:

$${{\text{h}}}^{2}=\frac{{\upsigma }_{{\text{a}}}^{2}}{{\upsigma }_{{\text{p}}}^{2}},$$

and estimates of the proportion of variance explained by maternal environmental effects (${{\text{m}}}^{2}$), when significantly different from zero, were calculated based on:

$${{\text{m}}}^{2}=\frac{{\upsigma }_{{\text{m}}}^{2}}{{\upsigma }_{{\text{p}}}^{2}},$$

where ${\upsigma }_{{\text{p}}}^{2}$ is the phenotypic variance, calculated based on ${\upsigma }_{{\text{p}}}^{2}={\upsigma }_{{\text{a}}}^{2}+{\upsigma }_{{\text{e}}}^{2}$ or ${\upsigma }_{{\text{p}}}^{2}={\upsigma }_{{\text{a}}}^{2}+{\upsigma }_{{\text{m}}}^{2}+{\upsigma }_{{\text{e}}}^{2}$ when ${\upsigma }_{{\text{m}}}^{2}$ was significantly different from zero.

Estimates of the genetic coefficient of variation (${\text{GCV}}$) were calculated as:

$${\text{GCV}}=\left|\frac{\sqrt{{\upsigma }_{{\text{a}}}^{2}}}{\upmu }\right|.$$

For ln(variance), the ${\text{GCV}}$ was calculated as $\sqrt{{\upsigma }_{{\text{a}}}^{2}}$, because the ln-transformation implicitly assumes an exponential model. Therefore, $\sqrt{{\upsigma }_{{\text{a}}}^{2}}$ has no units and division by $\upmu$ is redundant [38, 43].

Genetic correlations were estimated to investigate the relationship among traits. The correlations were estimated with bivariate analyses using the corresponding linear animal models (without or with maternal environmental effect, depending on its significance) described above for each trait. Estimates of genetic correlations (${{\text{r}}}_{{\text{a}}}$) were calculated as:

$${{\text{r}}}_{{{\text{a}}}_{12}}=\frac{{\upsigma }_{{{\text{a}}}_{12}}}{{\upsigma }_{{{\text{a}}}_{1}}^{2}{\upsigma }_{{{\text{a}}}_{2}}^{2}},$$

where ${{\text{r}}}_{{{\text{a}}}_{12}}$ is the genetic correlation between trait 1 and trait 2, ${\upsigma }_{{{\text{a}}}_{12}}$ is the genetic covariance between trait 1 and trait 2, ${\upsigma }_{{{\text{a}}}_{1}}^{2}$ is the additive genetic variance of trait 1, and ${\upsigma }_{{{\text{a}}}_{2}}^{2}$ is the additive genetic variance of trait 2.

## Results

### Resilience indicators

#### Heritabilities, GCV, and maternal environmental effects

Estimates of variance components, heritabilities, maternal environmental effects, and GCV of the resilience indicators ln(variance), skewness, and autocorrelation based on average batch production for the two periods and different intervals are in Table 2 for White and Table 3 for Brown. Overall, heritability estimates were slightly higher for White than for Brown, ranging from 0.02 to 0.12 for White and from 0.02 to 0.08 for Brown. Heritability estimates for ln(variance) were higher than those for skewness and autocorrelation, and ranged from 0.07 to 0.12 for White and from 0.04 to 0.08 for Brown. Remarkably, for the 83-end period, the estimate of additive genetic variance for ln(variance) was twice as high for White than for Brown. Heritability estimates for skewness ranged from 0.04 to 0.06 for the 25–83 period for White (for the 83-end period, it was not significantly different from zero) and from 0.02 to 0.04 for Brown. Heritability estimates for autocorrelation ranged from 0.02 to 0.06 for White and from 0.02 to 0.05 for Brown. Generally, heritability estimates were lower when using longer intervals for ln(variance) and autocorrelation, but higher for skewness. Of the 18 heritability estimates in total for both lines, five estimates of the 83-end were not significantly different from zero (one based on 1-week interval, two based on the 2-week-intervals, and two based on the 3-week-intervals). However, most of the resilience indicators were heritable.

**Table 2:** Estimates of genetic parameters of resilience indicators based on average batch production for White

Trait	Period	Interval	${{\varvec{\upsigma}}}_{\mathbf{a}}^{2}$ (SE)	${{\varvec{\upsigma}}}_{\mathbf{m}}^{2}$ (SE)	${{\varvec{\upsigma}}}_{\mathbf{e}}^{2}$ (SE)	${{\varvec{\upsigma}}}_{\mathbf{p}}^{2}$ (SE)	${\mathbf{h}}^{2}$ (SE)	${\mathbf{m}}^{2}$ (SE)	GCV
ln(variance)	25–83	1 week	0.07 (0.01)	NS	0.61 (0.01)	0.68 (0.01)	0.10 (0.01)	NS	0.26
		2 weeks	0.07 (0.01)	NS	0.78 (0.01)	0.86 (0.01)	0.08 (0.01)	NS	0.26
		3 weeks	0.07 (0.01)	NS^a^	0.89 (0.01)	0.97 (0.01)	0.07 (0.01)	NS^a^	0.26
	83-end	1 week	0.14 (0.01)	NS^a^	1.01 (0.01)	1.15 (0.01)	0.12 (0.01)	NS^a^	0.37
		2 weeks	0.16 (0.01)	NS	1.28 (0.01)	1.44 (0.01)	0.11 (0.01)	NS	0.40
		3 weeks	0.15 (0.02)	NS	1.60 (0.02)	1.75 (0.02)	0.09 (0.01)	NS	0.39
Skewness	25–83	1 week	0.04 (0.01)	NS	0.92 (0.01)	0.96 (0.01)	0.04 (0.01)	NS	0.13
		2 weeks	0.05 (0.01)	NS	0.87 (0.01)	0.92 (0.01)	0.05 (0.01)	NS	0.23
		3 weeks	0.04 (0.01)	NS^a^	0.72 (0.01)	0.76 (0.01)	0.06 (0.01)	NS^a^	0.30
	83-end	1 week	${\sigma }_{a}^{2}$ not significantly different from zero						
		2 weeks	${\sigma }_{a}^{2}$ not significantly different from zero						
		3 weeks	${\sigma }_{a}^{2}$ not significantly different from zero						
Autocorrelation	25–83	1 week	0.003 (0.0004)	NS	0.05 (0.0004)	0.05 (0.0004)	0.06 (0.01)	NS	0.26
		2 weeks	0.002 (0.0003)	NS	0.06 (0.001)	0.06 (0.001)	0.03 (0.01)	NS	0.32
		3 weeks	0.002 (0.0003)	NS	0.07 (0.001)	0.07 (0.001)	0.02 (0.005)	NS	0.41
	83-end	1 week	0.003 (0.001)	NS	0.09 (0.001)	0.09 (0.001)	0.04 (0.01)	NS	0.46
		2 weeks	${\sigma }_{a}^{2}$ not significantly different from zero						
		3 weeks	${\sigma }_{a}^{2}$ not significantly different from zero						

Estimates of variance components (${\upsigma }^{2}$), heritability (${{\text{h}}}^{2}$), maternal environmental effect (${{\text{m}}}^{2}$), and genetic coefficient of variation (GCV) of the three resilience indicators ln(variance), skewness, and autocorrelation based on average batch production for different life periods and with different intervals with standard errors (SE) for White

NS: maternal environmental effect not significantly different from zero

^a^Tended to be significantly different from zero (i.e. 0.05 < p ≤ 0.10)

**Table 3:** Estimates of genetic parameters of resilience indicators based on average batch production for Brown

Trait	Period	Interval	${{\varvec{\upsigma}}}_{\mathbf{a}}^{2}$ (SE)	${{\varvec{\upsigma}}}_{\mathbf{m}}^{2}$ (SE)	${{\varvec{\upsigma}}}_{\mathbf{e}}^{2}$ (SE)	${{\varvec{\upsigma}}}_{\mathbf{p}}^{2}$ (SE)	${\mathbf{h}}^{2}$ (SE)	${\mathbf{m}}^{2}$ (SE)	GCV
ln(variance)	25–83	1 week	0.09 (0.01)	0.02 (0.004)	1.03 (0.01)	1.14 (0.01)	0.08 (0.01)	0.02 (0.004)	0.30
		2 weeks	0.10 (0.01)	0.03 (0.01)	1.31 (0.01)	1.44 (0.01)	0.07 (0.01)	0.02 (0.004)	0.32
		3 weeks	0.09 (0.01)	0.04 (0.01)	1.49 (0.01)	1.62 (0.01)	0.06 (0.01)	0.02 (0.004)	0.30
	83-end	1 week	0.07 (0.01)	NS	1.48 (0.01)	1.55 (0.01)	0.04 (0.01)	NS	0.26
		2 weeks	0.08 (0.01)	NS	1.81 (0.02)	1.89 (0.02)	0.04 (0.01)	NS	0.28
		3 weeks	${\sigma }_{a}^{2}$ not significantly different from zero^a^						
Skewness	25–83	1 week	0.02 (0.01)	0.01 (0.004)	1.23 (0.01)	1.26 (0.01)	0.02 (0.005)	0.01 (0.003)	0.12
		2 weeks	0.03 (0.01)	0.01 (0.004)	1.16 (0.01)	1.21 (0.01)	0.03 (0.01)	0.01 (0.003)	0.22
		3 weeks	0.03 (0.01)	0.01 (0.003)	0.97 (0.01)	1.01 (0.01)	0.03 (0.01)	0.01 (0.003)	0.31
	83-end	1 week	0.02 (0.003)	NS	0.45 (0.004)	0.47 (0.004)	0.04 (0.01)	NS	0.44
		2 weeks	${\sigma }_{a}^{2}$ not significantly different from zero						
		3 weeks	${\sigma }_{a}^{2}$ not significantly different from zero						
Autocorrelation	25–83	1 week	0.004 (0.001)	0.001 (0.0003)	0.07 (0.001)	0.08 (0.001)	0.05 (0.01)	0.02 (0.004)	0.18
		2 weeks	0.002 (0.0005)	0.001 (0.0003)	0.08 (0.001)	0.08 (0.001)	0.03 (0.01)	0.01 (0.003)	0.12
		3 weeks	0.003 (0.0005)	NS	0.08 (0.001)	0.08 (0.001)	0.03 (0.01)	NS	0.17
	83-end	1 week	0.003 (0.001)	NS	0.11 (0.001)	0.12 (0.001)	0.02 (0.005)	NS	0.42
		2 weeks	${\sigma }_{a}^{2}$ not significantly different from zero						
		3 weeks	${\sigma }_{a}^{2}$ not significantly different from zero						

Estimates of variance components (${\upsigma }^{2}$), heritability (${{\text{h}}}^{2}$), maternal environmental effect (${{\text{m}}}^{2}$), and genetic coefficient of variation (GCV) of the three resilience indicators ln(variance), skewness, and autocorrelation based on average batch production for different life periods and with different intervals with standard errors (SE) for Brown

NS: maternal environmental effect not significantly different from zero

^a^Tended to be significantly different from zero (i.e. 0.05 < p ≤ 0.10)

In spite of the rather low heritability estimates, estimates of GCV were high: GCV estimates for ln(variance) ranged from 0.26 to 0.40 for White and from 0.26 to 0.32 for Brown. GCV estimates for skewness ranged from 0.13 to 0.30 for White and from 0.12 to 0.44 for Brown. GCV estimates for autocorrelation ranged from 0.26 to 0.46 for White and from 0.12 to 0.42 for Brown. Thus, the resilience indicators showed high genetic variability.

Maternal environmental effects were estimated to be either absent or small (0.01 to 0.02). Remarkably, maternal environmental effects were absent for White but mostly present in the 25–83 period for Brown.

#### Similarity between resilience indicators based on different intervals

Genetic correlations were estimated to investigate the similarities between resilience indicators based on average batch production in the same life periods but computed using different intervals (see Table 4). Resulting estimates of genetic correlations were generally high to very high (≥ 0.68). Estimates of genetic correlations ranged from 0.95 to 0.999 for ln(variance), from 0.87 to 0.97 for skewness, and from 0.68 to 0.90 for autocorrelation. Overall, the genetic correlation estimates indicated that resilience indicators computed based on 1-, 2-, or 3-week intervals were genetically similar or even close to identical.

**Table 4:** Estimates of genetic correlations between resilience indicators based on average batch production but computed using different intervals

Trait	Period	Interval	1 week	2 weeks	3 weeks
ln(variance)	25–83	1 week	–	^*a*^	*0.98 (0.01)*
		2 weeks	^a^	-	^*a*^
		3 weeks	0.97 (0.01)	^a^	-
	83-end	1 week	–	*0.99 (0.01)*	*0.95 (0.07)*
		2 weeks	0.99 (0.003)	–	^*a*^
		3 weeks	0.98 (0.01)	0.999 (0.004)	–
Skewness	25–83	1 week	–	*0.94 (0.03)*	*0.90 (0.05)*
		2 weeks	0.89 (0.02)	–	^*a*^
		3 weeks	0.87 (0.03)	0.97 (0.01)	–
	83-end	1 week	-	^*b*^	^*b*^
		2 weeks	^b^	-	^*b*^
		3 weeks	^b^	^b^	–
Autocorrelation	25–83	1 week	–	*0.84 (0.04)*	*0.84 (0.04)*
		2 weeks	0.90 (0.03)	–	*0.68 (0.06)*
		3 weeks	0.86 (0.05)	^a^	–
	83-end	1 week	–	^*b*^	^*b*^
		2 weeks	^b^	-	^*b*^
		3 weeks	^b^	^b^	–

Estimates of genetic correlations with standard errors (in parentheses) between the resilience indicators ln(variance), skewness, and autocorrelation based on average batch production for the same life periods, but with different intervals for White (below the diagonal) and Brown (above the diagonal, *italic*)

^a^Analysis did not converge

^b^Not tested, because one or both traits were not heritable (i.e. not significantly different from zero)

Given the lower estimates of residual variance and higher estimates of heritability for resilience indicators based on 1-week-intervals compared to the 2-week- and 3-week-intervals, as well as the high genetic correlation estimates between the resilience indicators for different intervals, results for resilience indicators based on 1-week-intervals will be reported in the remainder, in order to keep focus and to improve readability.

#### Similarity between the resilience indicators for different life periods

Genetic correlations were estimated to investigate the similarities between resilience indicators based on average batch production for the same intervals, but in the two life periods (see Table 5). Resulting estimates were moderate to very high ( ≥|0.46|), especially for ln(variance) and autocorrelation (0.73–0.80), while the estimate for skewness was moderately negative for Brown (-0.46). Unfortunately, the latter estimate could not be confirmed for White, because the estimate of heritability for skewness for the 83-end period was not significantly different from zero for White and was, therefore, not tested. In general, however, the resilience indicators estimated for the two periods were found to have a similar genetic composition.

**Table 5:** Estimates of genetic correlations between resilience indicators based on average batch production for different life periods

trait	Period	25–83	83-end
ln(variance)	25–83	–	*0.76 (0.07)*
	83-end	0.80 (0.04)	–
Skewness	25–83	–	− *0.46 (0.13)*
	83-end	^a^	–
Autocorrelation	25–83	–	*0.80 (0.08)*
	83-end	0.73 (0.07)	–

Estimates of genetic correlations with standard errors (in parentheses) between the resilience indicators ln(variance), skewness, and autocorrelation based on average batch production for 1-week-intervals, but for different life periods for White (below the diagonal) and Brown (above the diagonal, *italic*)

^a^Not tested, because one or both traits were not heritable (i.e. not significantly different from zero)

#### Similarity between the different resilience indicators

Estimates of genetic correlations between the three types of resilience indicators for the same life period were estimated (see Table 6). Resulting estimates were low to moderate (|0.01|–|0.67|), and their direction and magnitudes were mostly similar for White and Brown. The ln(variance) and autocorrelation were estimated to be lowly (or not) genetically correlated, except for the 83-end period for Brown (0.42). Skewness had moderate genetic correlation estimates with both ln(variance) and autocorrelation, although it was estimated to be negatively correlated with both for the 25–83 period, while it was estimated to be positively correlated with autocorrelation for the 83-end period for Brown (ln(variance) for White was not tested). Overall, the low to moderate genetic correlation estimates between the resilience indicators indicate that they capture different aspects of the genetic variation of EP deviations.

**Table 6:** Estimates of genetic correlations between resilience indicators based on average batch production

Trait	Period	ln(variance)	Skewness	Autocorrelation
ln(variance)	25–83	–	− *0.67 (0.11)*	*0.14 (0.10)*
	83-end	–	*0.65 (0.09)*	*0.42 (0.11)*
Skewness	25–83	− 0.46 (0.07)	–	− *0.55 (0.15)*
	83-end	^a^	–	*0.36 (0.13)*
Autocorrelation	25–83	− 0.01 (0.08)	− 0.21 (0.09)	–
	83-end	0.18 (0.08)	^a^	–

Estimates of genetic correlations with standard errors (in parentheses) between the resilience indicators ln(variance), skewness, and autocorrelation based on average batch production for the same life periods with 1-week-intervals for White (below the diagonal) and Brown (above the diagonal, *italic*)

^a^Not tested, because one or both traits were not heritable (i.e. not significantly different from zero)

#### Similarity between resilience indicators derived using different expected productions

**Fig. 2:**
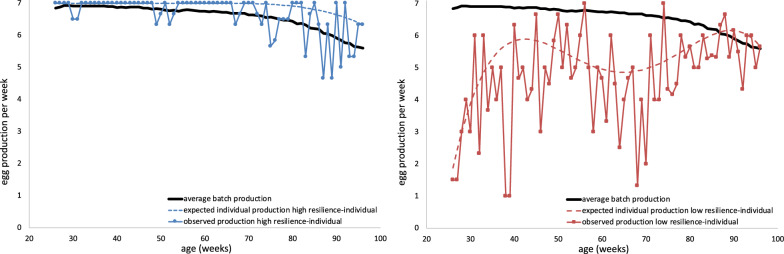
Example of observed and expected egg production of a highly resilient individual and a lowly resilient individual. Observed and expected 1-week-interval egg production of a selected highly resilient individual (left, blue, circles) and a selected lowly resilient individual (right, red, squares) from the same batch. Expected egg production was set to the average batch production (solid black line) or was based on the egg production curve of each individual that was estimated with a 4th order polynomial quantile regression using a 0.7 quantile fitted through 1-week-interval egg production observations during the production cycle between 25 weeks of age and the end of the individual’s life (dashed lines). The individuals shown were selected based on their low (around 10th percentile; high resilience) or high (around 90th percentile; low resilience) ln(variance) between 25 weeks of age and the end of life

Estimates of variance components, heritabilities, maternal environmental effects, and GCV of the resilience indicators based on expected individual production for the 25–83 and 83-end periods with 1-week-intervals are in Table 7 for White and Table 8 for Brown. Figure 2 illustrates two examples of expected individual EP based on average batch production and based on own production. In general, the resilience indicators based on the expected individual production showed similar estimates of variance components, heritabilities, maternal environmental effects, and GCV as the resilience indicators based on average batch production. Although, overall, estimates of environmental variance of resilience indicators based on expected individual production were slightly lower, possibly due to the larger maternal environmental effects, their heritability estimates were slightly higher compared to those based on deviations from average batch production. Heritability estimates for skewness in the 83-end period based on individual expected production were at least twice as high as corresponding estimates based on average batch production. Therefore, skewness appeared to be sensitive to the definition of the expected production, but ln(variance) and autocorrelation were hardly affected.

**Table 7:** Estimates of genetic parameters of resilience indicators based on expected individual production for White

Trait	Period	${{\varvec{\upsigma}}}_{\mathbf{a}}^{2}$ (SE)	${{\varvec{\upsigma}}}_{\mathbf{m}}^{2}$ (SE)	${{\varvec{\upsigma}}}_{\mathbf{e}}^{2}$ (SE)	${{\varvec{\upsigma}}}_{\mathbf{p}}^{2}$ (SE)	${\mathbf{h}}^{2}$ (SE)	${\mathbf{m}}^{2}$ (SE)	GCV
ln(variance)	25–83	0.07 (0.01)	0.004 (0.002)	0.52 (0.01)	0.59 (0.01)	0.12 (0.01)	0.01 (0.003)	0.26
	83-end	0.14 (0.01)	0.01 (0.003)	0.92 (0.01)	1.07 (0.01)	0.13 (0.01)	0.01 (0.003)	0.37
Skewness	25–83	0.10 (0.01)	0.01 (0.003)	0.91 (0.01)	1.02 (0.01)	0.10 (0.01)	0.01 (0.003)	0.12
	83-end	0.05 (0.01)	NS	0.50 (0.01)	0.50 (0.005)	0.09 (0.01)	NS	0.27
Autocorrelation	25–83	0.001 (0.0002)	NS	0.03 (0.0003)	0.03 (0.0002)	0.03 (0.01)	NS	0.14
	83-end	0.003 (0.0005)	NS	0.08 (0.001)	0.08 (0.001)	0.04 (0.01)	NS	0.78

Estimates of variance components (${\upsigma }^{2}$), heritability (${{\text{h}}}^{2}$), maternal environmental effect (${{\text{m}}}^{2}$), and genetic coefficient of variation (GCV) of the three resilience indicators ln(variance), skewness, and autocorrelation based on expected individual production for the selected life periods with 1-week intervals with standard errors (SE) for White

NS: maternal environmental effect not significantly different from zero

**Table 8:** Estimates of genetic parameters of resilience indicators based on expected individual production for Brown

Trait	Period	${{\varvec{\upsigma}}}_{\mathbf{a}}^{2}$ (SE)	${{\varvec{\upsigma}}}_{\mathbf{m}}^{2}$ (SE)	${{\varvec{\upsigma}}}_{\mathbf{e}}^{2}$ (SE)	${{\varvec{\upsigma}}}_{\mathbf{p}}^{2}$ (SE)	${\mathbf{h}}^{2}$ (SE)	${\mathbf{m}}^{2}$ (SE)	GCV
ln(variance)	25–83	0.11 (0.01)	0.02 (0.005)	1.09 (0.01)	1.22 (0.01)	0.09 (0.01)	0.02 (0.004)	0.33
	83-end	0.05 (0.01)	0.01 (0.01)	1.70 (0.02)	1.76 (0.02)	0.03 (0.01)	0.01 (0.003)	0.22
Skewness	25–83	0.11 (0.01)	0.02 (0.005)	1.26 (0.01)	1.36 (0.01)	0.08 (0.01)	0.01 (0.004)	0.17
	83-end	0.02 (0.003)	0.004 (0.002)	0.46 (0.005)	0.48 (0.004)	0.04 (0.01)	0.01 (0.004)	0.31
Autocorrelation	25–83	0.003 (0.001)	0.001 (0.0002)	0.07 (0.001)	0.07 (0.001)	0.05 (0.008)	0.01 (0.003)	0.25
	83-end	0.003 (0.001)	NS	0.11 (0.001)	0.11 (0.001)	0.03 (0.01)	NS	0.55

Estimates of variance components (${\upsigma }^{2}$), heritability (${{\text{h}}}^{2}$), maternal environmental effect (${{\text{m}}}^{2}$), and genetic coefficient of variation (GCV) of the three resilience indicators ln(variance), skewness, and autocorrelation based on expected individual production for the selected life periods with 1-week intervals with standard errors (SE) for Brown

NS: maternal environmental effect not significantly different from zero

Genetic correlations were estimated to investigate the similarities between resilience indicators based on average batch production and those based on expected individual production for the same periods and the same intervals (see Table 9). Resulting estimates ranged from 0.001 to 0.99 and were all positive. Genetic correlation estimates were very high (≥ 0.92) for the 83-end period for all resilience indicators and also for ln(variance) for the 25–83 period (≥ 0.95). Thus, these traits captured the same genetic variation and were mostly independent of the method used to calculate the expected EP. However, genetic correlation estimates were low (0.001 and 0.27) for the 25–83 period for skewness and moderate (0.62 and 0.73) for autocorrelation for the 25–83 period. Thus, overall, expected production did not have a major influence on ln(variance) for the 25–83 period and on all resilience indicators for the 83-end period, it had a minor influence on autocorrelations for the 25–83 period but a large effect on skewness for the 25–83 period.

**Table 9:** Estimates of genetic correlations between resilience indicators based on average batch production and expected individual production

Trait	Period	White	*Brown*
ln(variance)	25–83	0.95 (0.01)	*0.98 (0.01)*
	83-end	0.996 (0.002)	*0.99 (0.01)*
Skewness	25–83	0.27 (0.08)	*0.001 (0.14)*
	83-end	^a^	*0.99 (0.01)*
Autocorrelation	25–83	0.62 (0.06)	*0.73 (0.06)*
	83-end	0.92 (0.02)	*0.97 (0.02)*

Estimates of genetic correlations with standard errors (in parentheses) between the resilience indicators ln(variance), skewness, and autocorrelation based on average batch production and expected individual production for the same life periods with 1-week-intervals for White and Brown (*italic*)

^a^Not tested, because one or both traits were not heritable (i.e. not significantly different from zero)

### Resilience indicators and egg production traits

The genetic relationships between the resilience indicators and the EP traits were investigated by estimating their genetic correlations (see Table 10). Resulting estimates were low to very high (|0.03|–|0.99|). Early EP, which is the period of EP that was not taken into account for determining the resilience indicators, had low genetic correlation estimates ( ≤|0.21|) with the resilience indicators. ln(variance) had favorable genetic correlation estimates with the other EP traits: high with EP in the 25–83 period (|0.72|–|0.88|) and moderate to high with EP in the 83-end period (|0.42|–|0.81|). Skewness in White had low, but favorable genetic correlation estimates ( ≤|0.33|) with EP traits. However, in Brown, skewness had moderate to high favorable genetic correlation estimates (|0.49|–|0.70|) with EP in the 25–83 period and very high unfavorable genetic correlation estimates ( ≥|0.91|) with EP in the 83-end period. In general, autocorrelation had low to moderate genetic correlation estimates (|0.02|–|0.42|) with the EP traits, which were mostly in the favorable direction. In summary, the resilience indicators were mostly favorably genetically correlated with EP traits, but the extent of these correlations varied.

**Table 10:** Estimates of genetic correlations between resilience indicators and egg production traits

		Period	Full	Early	25–83	83-end
White	ln(variance)	25–83	− 0.82 (0.02)	0.05 (0.05)	− 0.72 (0.04)	− 0.88 (0.02)
		83-end	− 0.62 (0.05)	0.10 (0.05)	− 0.43 (0.06)	− 0.81 (0.03)
	Skewness	25–83	0.11 (0.08)	− 0.15 (0.06)	− 0.05 (0.09)	0.33 (0.08)
		83-end	^a^	^a^	^a^	^a^
	Autocorrelation	25–83	− 0.04 (0.07)	− 0.06 (0.06)	0.25 (0.09)	− 0.23 (0.07)
		83-end	− 0.16 (0.08)	− 0.07 (0.07)	− 0.03 (0.09)	− 0.22 (0.08)
*Brown*	ln(variance)	25–83	− *0.84 (0.03)*	− *0.14 (0.07)*	− *0.83 (0.04)*	− *0.77 (0.04)*
		83-end	− *0.46 (0.08)*	*0.18 (0.07)*	− *0.42 (0.09)*	− *0.59 (0.07)*
	Skewness	25–83	*0.53 (0.14)*	− *0.21 (0.11)*	*0.49 (0.15)*	*0.70 (0.11)*
		83-end	− *0.95 (0.04)*	*0.06 (0.07)*	− *0.91 (0.05)*	− *0.99 (0.02)*
	Autocorrelation	25–83	*0.22 (0.10)*	− *0.12 (0.08)*	*0.38 (0.11)*	− *0.02 (0.09)*
		83-end	− *0.25 (0.10)*	− *0.04 (0.09)*	− *0.11 (0.11)*	− *0.42 (0.09)*

Estimates of genetic correlations with standard errors (in parentheses) between the resilience indicators ln(variance), skewness, and autocorrelation based on average batch production for the same life periods with 1-week-intervals and egg production traits for the full production cycle (i.e. all eggs between start and end), the early production cycle (i.e. between start and 25 weeks of age), the 'traditional' production cycle (i.e. between 25 and 83 weeks of age), and the time period after the 'traditional' production cycle (i.e. between 83 weeks of age and end) for White (top) and Brown (bottom, *italic*)

^a^Not tested, because the resilience indicator trait was not heritable (i.e. not significantly different from zero)

### Resilience indicators and antibody traits

Genetic relationships between the resilience indicators and the antibody traits were investigated by estimating their genetic correlations (see Table 11). Resulting estimates were low to moderate (|0.02|–|0.56|) with large SE, especially for Brown because the resilience indicators and the antibody traits were measured on different individuals. In summary, the evaluated resilience indicators and the antibody traits were found to be mostly not genetically correlated.

**Table 11:** Estimates of genetic correlations between resilience indicators and antibody traits

	Period	White		Brown	
		IgM	IgG	IgM	IgG
ln(variance)	25–83	− 0.09 (0.11)	0.06 (0.17)	− *0.03 (0.30)*	− *0.27 (0.32)*
	83-end	− 0.12 (0.12)	− 0.02 (0.18)	− *0.21 (0.34)*	− *0.24 (0.39)*
Skewness	25–83	0.08 (0.13)	− 0.18 (0.19)	− *0.16 (0.42)*	*0.16 (0.48)*
	83-end	^a^	^a^	*0.28 (0.34)*	− *0.09 (0.39)*
Autocorrelation	25–83	0.16 (0.12)	0.21 (0.18)	− *0.09 (0.31)*	− *0.15 (0.33)*
	83-end	0.15 (0.15)	0.13 (0.21)	*0.03 (0.42)*	*0.56 (0.42)*

Estimates of genetic correlations with standard errors (in parentheses) of the resilience indicators ln(variance), skewness, and autocorrelation based on average batch production for the same life periods with 1-week-intervals and the antibody traits keyhole limpet hemocyanin (KLH)-binding IgM natural antibodies and KLH-binding IgG natural antibodies for White and Brown (*italic*)

^a^Not tested, because the resilience indicator trait was not heritable (i.e. not significantly different from zero)

## Discussion

Breeding for improved resilience is a promising strategy to decrease labor and health costs of livestock and to prevent reduced production [2, 3, 27], but to date, it has not been tested because of a lack of suitable resilience indicator(s). During the last decade, technological developments to collect and analyze big data have opened the way to develop resilience indicators [21]. Longitudinal data can now be easily collected on individuals and used to investigate new phenotypes, including resilience indicators. Three such resilience indicators were proposed by Berghof et al. [2] based on deviations between expected and observed production, i.e. ln(variance) of deviations, skewness of deviations, and autocorrelation of deviations. Such deviations can occur as a response to disease, heat, or management changes (e.g. feed composition). This study investigated the effect of different interval lengths and age periods on genetic characteristics of these resilience indicators based on longitudinally observed day-to-day deviations of EP in laying hens. Moreover, this study investigated possible pleiotropic effects (i.e. trade-offs) between resilience and production and between resilience and immunity. Our results provide good indications for the definition of new resilience indicators for chicken breeding.

### Resilience indicators

The heritability estimates for the three resilience indicators (0.02–0.12) analyzed here were lower than (comparable) resilience indicators previously reported in the few studies that were based on longitudinal data [22–25, 27, 28, 31], but were in line with those from studies investigating uniformity in livestock (see Iung et al. [44] for an overview). Bedere et al. [28], using crossbred chickens, which are related to the chicken population used here and with similar phenotypes, reported heritabilities that ranged from 0.01 to 0.21 for the three resilience indicators based on deviations from average batch EP. In dairy cows and pigs, heritability estimates for resilience traits are generally higher [22–25, 31], which is possibly due to the larger number of observations per animal; i.e. more observations increase heritability estimates [2]. We observed a similar trend when comparing resilience indicators computed based on 1-week-, 2-week, and 3-week-intervals, since the number of observations per animal decreases as the interval size increases. However, it should be noted that, in a previous study in purebred chickens [27], we found relatively high heritability estimates of about 0.10 for the three resilience indicators based on deviations of body weight (BW) with only five to eight observations per animal, which does not support the previous argument that a larger number of observations increases heritabilities. In the current study, the median numbers of observations during the total production cycle were 74 for White and 68 for Brown for 1-week-intervals, 37 and 34 for 2-week-intervals, and 24 and 22 for 3-week-intervals per individual, respectively, and we used a minimum of five observations per individual. Heritability estimates for resilience indicators based on EP deviations were expected to be higher than heritability estimates of the resilience indicators based on BW deviations, given the number of observations per animal. A possible explanation for this ‘inconsistency’ probably lies in the differences in nature of these deviations. Egg production can be considered as a ‘maximum trait’, meaning that breeding programs aim at reaching the maximum phenotype of one egg per day, which is the biological limit, and positive deviations in EP can only be the consequence of an additional egg laid the day before (as explained in ‘Methods’). Moreover, EP is binary, making the interpretation of resilience indicators based on EP challenging (see also Doekes et al. [32]). In contrast, BW can be considered as an ‘optimum trait’, i.e. a trait that approximates a normal distribution and might have a desired optimum value or range for all livestock species. For such a trait, both negative and positive deviations can occur (the latter likely due to some compensatory mechanism during recovery from a disturbance), which might result in higher heritabilities. Similar ‘optimum traits’ are eggshell strength and egg weight, but these traits are currently not collected on the individual and longitudinal scale (i.e. daily measurements on each egg). However, we hypothesize that these ‘optimum traits’ with both positive and negative deviations are better phenotypes on which to base longitudinal production data-based resilience indicators and have higher heritabilities than ‘maximum traits’.

To assess how this ‘maximum trait’ with its binary nature on a daily scale impacts the genetic parameters of the resilience indicators, we investigated EP deviations computed based on different intervals, i.e. 1-week-, 2-week-, and 3-week-intervals. We reasoned that, due to the long-term intensive selection for EP in these populations, few fluctuations should occur during short intervals, which would heavily skew the deviation data. For this reason, we excluded daily observations as an option to determine the deviations. Moreover, because EP was recorded on a one- to four-day interval, we chose to use the three intervals mentioned above. We hypothesized that, on the one hand, a shorter interval would result in a higher estimate of heritability because of the larger number of observations per animal, but, that on the other hand, a longer interval would provide more opportunity for variation to occur when EP is very close to one egg per day. However, it can also be argued that shorter intervals better represent the volatility of (small) disturbances in an environment. Nevertheless, the results show convincingly that the resilience indicators derived using the three interval lengths are genetically highly correlated and, thus, hardly/not affected by interval length. Therefore, we selected resilience indicators based on 1-week-intervals as the most promising, because their environmental variance was the smallest and, consequently, their heritability estimates were the highest.

We also investigated two periods (25–83 and 83-end) during the productive life of chickens because we reasoned that with the only recent focus of breeding programs on a prolonged productive life (after 83 weeks of age), there would be more EP fluctuations after than before 83 weeks of age (as explained in ‘Methods’). Indeed, although estimates of the genetic correlations between resilience indicators based on the 25–83 and 83-end periods were not equal to 1, they were moderate to high. Thus, determining the resilience indicators for both these life periods is relevant for the improvement of resilience throughout the whole production cycle. Genomic selection provides the possibility of including these resilience indicators in breeding programs, especially for the 83-end period, since the observations on selection candidates will come in too late to be used in their selection.

The longitudinal data allowed us to estimate the genetic correlations between resilience indicators computed based on deviations from average batch production versus expected individual production, similar to Poppe et al. [23–26]. Genetic correlation estimates for the 25–83 period were very high for ln(variance), moderate for autocorrelation, and low for skewness. Thus, phenotypes for ln(variance) and autocorrelation do not depend (so much) on the absolute level of the expected production, but on the actual fluctuations of the deviations. Skewness, on the other hand, is, by definition, strongly influenced by outliers and, thus, more affected by the expected production. These results are also in line with those of Berghof et al. [27] and Poppe et al. [23], who used different methods to set the expected production and found that this did not affect ln(variance) and autocorrelation, but did affect skewness [23, 27]. This also means that, compared to skewness, ln(variance) and autocorrelation can be more easily compared between studies that use different approaches to compute deviations. The reason for the very high genetic correlation estimates for the 83-end period between the two methods investigated here should probably be sought in the decrease in EP and its greater variation at the end of the chicken’s life. Although both the expected individual production and the average batch production can underestimate deviations in their own way (see also Bedere et al. [28]), this does not seem to have a major effect on ln(variance) and autocorrelation, which is a direct consequence of the characteristics of these indicators.

It is important to realize that for these resilience indicators, it is assumed that the deviations actually occurred as a consequence of environmental disturbances rather than being part of a ‘normal’ performance trajectory. We deem this assumption likely because there is no indication that average batch EP shows structural fluctuations in EP (e.g. Fig. 2). The generally high genetic correlation estimates between resilience indicators computed using different methods support this idea (e.g. Berghof et al. [27], Poppe et al. [23], and our results). Therefore, individual deviations are likely consequences of randomly occurring disturbances. In addition, the resilience indicators investigated here are mostly independent of the absolute level of the trait and only based on fluctuations in deviations between observed and expected production over time. Thus, a structural fluctuation in EP, as part of a normal EP curve, is expected to minimally affect the resilience indicators. Nevertheless, any information on the nature of the disturbances that occurred would be valuable information to further define and verify the resilience indicators, but this was not available for the chicken populations studied here.

In this study, we selected the resilience indicators based on having the highest heritability estimates and high genetic correlation estimates with other indicators. It should be noted that, although heritability estimates were relatively low in general, estimates of GCV were high, which indicates a high genetic variability and is consistent with other reports in the literature (see overviews in Mulder et al. [43], Hill and Mulder [38], and Iung et al. [44]). Moreover, our study is the first to report significant, although marginal, maternal environmental effects on resilience indicators. However, due to the non-specific nature of the fluctuations, the physiology of the maternal effects remains unclear. In addition, as consistently reported in other studies, estimates of genetic correlations between the three resilience indicators were low to moderate, suggesting that they cover different genetic aspects of resilience [23, 24, 27, 28]. Thus, the resilience indicators based on EP that have the highest potential for breeding programs are those based on 1-week deviations from either average batch EP or individual EP for the 25–83 and 83-end periods.

### Resilience indicators and egg production traits

Trade-offs between resilience and production can be expected based on resource allocation theory, which states that an organism’s limited resources are allocated to its different energy requirements resulting in trade-offs between these requirements [45–47]. To investigate the presence of trade-offs between resilience indicators and EP traits at the genetic level, genetic correlations between resilience indicators and EP were estimated.

Resulting estimates of genetic correlations were mostly favorable or low and, therefore, trade-offs seem to be almost absent or, in some cases, even synergetic. ln(variance) had moderate to high favorable genetic correlation estimates with EP after 25 weeks of age: the higher the EP, the smaller the number of days with no egg, and thus the less fluctuation. However, this is also expected to hold for the opposite phenotype, i.e. low EP results in less fluctuation (see also Doekes et al. [32]). However, selection on EP has apparently been so strong and consistent, that there are nearly no chickens with an extremely low EP. For skewness for the 25–83 period, the genetic correlation estimates were low, but favorable, similar to those for ln(variance). Unexpectedly, skewness for the 83-end period was estimated to be unfavorably and very highly correlated with EP traits in Brown. Thus, a high negative skewness of deviations (i.e. fewer eggs than average) genetically correlates with higher production, which is counter intuitive and, to date, we do not have a conclusive explanation for this result. One possibility of this unexpected behavior of skewness is that it is under the relatively strong influence of outliers. Unfortunately, skewness for the 83-end period was not significantly heritable in White, and we cannot confirm this observation in both lines. This result, however, adds to the questionable usefulness of skewness as a resilience indicator [23, 24, 27, 28]. For autocorrelation, a similar phenomenon was observed, although to a lesser extent: autocorrelation for the 25–83 period was estimated to be unfavorably but weakly genetically correlated with EP for the 25–83 period, but the estimate of the genetic correlation of autocorrelation for the 83-end period was favorable and weak to moderate with EP for the 83-end period. In addition, it will be of great interest to investigate the genetic correlation of these resilience indicators with production traits that are different from the production trait that was used to determine the resilience indicators, for example, to investigate the relationship between resilience indicators based on deviations of eggshell strength with EP traits. Nevertheless, the severity of the disturbances was probably too low to show trade-offs according to the resource theory, and, thus, selection for these resilience indicators can be implemented into breeding programs of purebred chickens without strong effects on EP.

### Resilience indicators and antibody traits

Higher levels of KLH-binding NAb at 20 weeks of age in chickens have been shown to be phenotypically associated with lower mortality later in life [48, 49] and with improved immunity [50, 51]. Moreover, chickens selected for high KLH-binding NAb levels had lower mortality after *E. coli*-infection at a young age compared to unselected chickens and chickens selected for low KLH-binding NAb levels [52]. Thus, NAb levels show potential as indicators of disease resistance, since they are assumed to protect animals and thereby reduce their sensitivity to environmental disturbances, e.g. disease. NAb could also be genetically correlated with (disease) resilience and could, thus, be used as resilience indicators (or vice versa).

However, in our study, genetic correlations between the resilience indicators and KLH-binding NAb at about 16 weeks of age (for disease resistance) were estimated to be low, which means that they capture other genetic aspects of health. In addition, a genome-wide association study for resilience indicators based on average batch EP showed no overlap in associated genomic regions with KLH-binding NAb in Brown [32]. Furthermore, genetic variants in the *toll-like receptor family member 1A* (*TLR1A*) gene, which is a major quantitative trait locus for KLH-binding NAb [34] in White, did not significantly explain variation for the resilience indicators (*TLR1A* as fixed effect in the model; results not shown). Similar to Berghof et al. [27], the lack of disease challenges in the biosecure breeding nucleus environment likely resulted in very limited fluctuations in production due to disease. In contrast, high-challenge environments are generally reported to result in more genetic variation in resistance or resilience than normal environments [53, 54]. Although estimated breeding values of ln(variance) were predictive of lesion scores after avian pathogenic *Escherichia coli* challenge in chickens, they were not predictive of mortality [27]. In addition, in pigs, the level of KLH-binding NAb was not significantly genetically correlated to resilience indicators based on the variance of the deviations of daily feed intake or of daily duration at the feeder, which were predictive of mortality, in a ‘natural disease challenge environment’ [31, 55]. Thus, it seems that also in challenge environments, the resilience indicators are only weakly related to the level of NAb, but more challenge studies are needed to investigate this, although they are costly and ethically controversial. The absence of unfavorable genetic correlations shows that selection to improve resilience using resilience indicators based on EP does not negatively affect immunity and, thus, could be complementary in breeding for improved resilience and health.

### Future directions

In this study, we examined different variants of potential resilience indicators previously analyzed [2, 23]. ln(variance) or similar traits related to variance of a phenotype gave consistently good results in several studies on different livestock species and traits [22–25, 27, 31]. However, the resilience indicators, skewness and autocorrelation, can be improved. More resilient animals are expected to have a more uniform production with fewer and smaller deviations compared to less resilient animals, and to have a skewness and an autocorrelation of deviations around zero compared to the population average [2]. Thus, both positive and negative values for skewness and autocorrelation are undesired, but a negative autocorrelation and/or a positive skewness are more favorable because they are indicative of a more resilient animal compared to individuals with a positive autocorrelation or negative skewness. It might be that due to the nature of skewness and autocorrelation, the results for these traits have been disappointing and sometimes confusing (also in this study), leading to the suggestion that they should be omitted in future analyses [23, 24, 27, 28]. In addition, the interpretation of the direction of resilience indicators probably needs more nuance, as illustrated by Doekes et al. [32]. Thus, before completely excluding autocorrelation and skewness from future studies, we suggest using autocorrelation and skewness based on absolute values of deviations (i.e. bringing the desired value to zero) or basing them on deviations for ‘optimum traits’ (e.g. eggshell strength or body weight and as shown for activity in Poppe et al. [26]). Moreover, future studies should consider the use of ‘optimum traits’ for determining resilience indicators, which would also allow investigation of the use of daily fluctuations compared to 1-week-interval fluctuations, because deviations for such traits are likely to be more normally distributed and not binary, as for daily EP. A major point of attention is that the resilience indicators should be studied in situations with known disturbances to verify their predictive properties to capture resilience, as was done by Putz et al. [31], Poppe et al. [25], and Poppe et al. [26]. These are exciting new steps in the search for informative resilience indicators for all livestock species.

## Conclusions

Breeding for improved resilience will reduce the sensitivity of livestock to environmental factors and, thereby, improve their health and economic profit. Although potential resilience indicators have been defined, how they are affected by underlying physiology requires more research. This study examined several variables (i.e. life periods, intervals, and different methods for expected production) that could affect resilience indicator traits based on EP, which is currently the most extensively measured longitudinal trait on individuals in layer chicken breeding programs. Based on the results, we conclude that the relevant resilience indicators based on EP are based on 1-week-intervals and for the periods 25–83 and/or 83-end. The three resilience indicators, i.e. ln(variance), skewness, and autocorrelation, are genetically different and, therefore, contain information on different aspects of resilience. The method for determining the expected EP (average batch production vs. expected individual production) did not have much influence on the results for ln(variance) and autocorrelation, which illustrates that these indicators capture fluctuations and are independent of expected EP. Estimates of genetic correlations between the resilience indicators and EP traits were mostly favorable or zero, which means that trade-offs are mostly absent. However, skewness for the 83-end period showed very strong and unfavorable genetic correlation estimates with EP in Brown, which requires further studies. Future research should also focus on the usefulness and interpretation of skewness and autocorrelation and on possible trade-offs between resilience indicators and other production traits in breeding programs. Finally, the analyzed resilience indicators did not show genetic correlations with the indicator of disease resistance, i.e. level of NAb, possibly because the chickens used were housed in a highly hygienic environment. Thus, although the investigated resilience indicators based on EP and level of NAb cannot be used as predictors of each other, they can be used in conjunction in breeding programs. In total, this study took several steps in defining and implementing indicators in breeding programs to improve the resilience of livestock.

### Supplementary Information

**Additional file 1: Table S1.** Estimates of genetic (below the diagonal) and phenotypic (above the diagonal, *italic*) correlations with standard errors (in parentheses) between the resilience indicators ln(variance), skewness, and autocorrelation based on average batch production for the same life periods, but with different intervals for White. **Table S2.** Estimates of genetic (below the diagonal) and phenotypic (above the diagonal, *italic*) correlations with standard errors (in parentheses) between the resilience indicators ln(variance), skewness, and autocorrelation based on average batch production for the same life periods, but with different intervals for Brown. **Table S3.** Estimates of genetic (below the diagonal) and phenotypic (above the diagonal, *italic*) correlations with standard errors (in parentheses) between the resilience indicators ln(variance), skewness, and autocorrelation based on average batch production for 1-week-intervals, but for different life periods for White. **Table S4.** Estimates of genetic (below the diagonal) and phenotypic (above the diagonal, *italic*) correlations with standard errors (in parentheses) between the resilience indicators ln(variance), skewness, and autocorrelation based on average batch production for 1-week-intervals, but for different life periods for Brown. **Table S5.** Estimates of genetic (below the diagonal) and phenotypic (above the diagonal, *italic*) correlations with standard errors (in parentheses) between the resilience indicators ln(variance), skewness, and autocorrelation based on average batch production for the same life periods with 1-week-intervals for White. **Table S6.** Estimates of genetic (below the diagonal) and phenotypic (above the diagonal, *italic*) correlations with standard errors (in parentheses) between the resilience indicators ln(variance), skewness, and autocorrelation based on average batch production for the same life periods with 1-week-intervals for Brown. **Table S7.** Estimates of genetic and phenotypic (*italic*) correlations with standard errors (in parentheses) between the resilience indicators ln(variance), skewness, and autocorrelation based on average batch production and expected individual production for the same life periods with 1-week-intervals for White and Brown. **Table S8.** Estimates of genetic characteristics for egg production traits: variance components (${\sigma }^{2}$), heritability (${h}^{2}$), and maternal environmental effect (${m}^{2}$) for the full production cycle (i.e. all eggs between start and end), the early production cycle (i.e. between start and 25 weeks of age), the 'traditional' production cycle (i.e. between 25 and 83 weeks of age), and the time period after the 'traditional' production cycle (i.e. between 83 weeks of age and end) with standard errors (SE) for White and Brown. **Table S9.** Estimates of genetic (below the diagonal) and phenotypic (above the diagonal, *italic*) correlations with standard errors (in parentheses) between egg production traits for the full production cycle (i.e. all eggs between start and end), the early production cycle (i.e. between start and 25 weeks of age), the 'traditional' production cycle (i.e. between 25 and 83 weeks of age), and the time period after the 'traditional' production cycle (i.e. between 83 weeks of age and end) for White. **Table S10.** Estimates of genetic (below the diagonal) and phenotypic (above the diagonal, *italic*) correlations with standard errors (in parentheses) between egg production traits for the full production cycle (i.e. all eggs between start and end), the early production cycle (i.e. between start and 25 weeks of age), the 'traditional' production cycle (i.e. between 25 and 83 weeks of age), and the time period after the 'traditional' production cycle (i.e. between 83 weeks of age and end) for Brown. **Table S11.** Estimates of genetic and phenotypic (*italic*) correlations with standard errors (in parentheses) between the resilience indicators ln(variance), skewness, and autocorrelation based on average batch production for the same life periods with 1-week-intervals and egg production traits for the full production cycle (i.e. all eggs between start and end), the early production cycle (i.e. between start and 25 weeks of age), the 'traditional' production cycle (i.e. between 25 and 83 weeks of age), and the time period after the 'traditional' production cycle (i.e. between 83 weeks of age and end) for White. **Table S12.** Estimates of genetic and phenotypic (*italic*) correlations with standard errors (in parentheses) between the resilience indicators ln(variance), skewness, and autocorrelation based on average batch production for the same life periods with 1-week-intervals and egg production traits for the full production cycle (i.e. all eggs between start and end), the early production cycle (i.e. between start and 25 weeks of age), the 'traditional' production cycle (i.e. between 25 and 83 weeks of age), and the time period after the 'traditional' production cycle (i.e. between 83 weeks of age and end) for Brown. **Table S13.** Estimates of genetic characteristics for the antibody traits: variance components (${\sigma }^{2}$), heritability (${h}^{2}$), and maternal environmental effect (${m}^{2}$) of the antibody traits keyhole limpet hemocyanin (KLH)-binding IgM natural antibody titer and KLH-binding IgG natural antibody titer with standard errors (SE) for White and Brown. **Table S14.** Estimates of genetic (below the diagonal) and phenotypic (above the diagonal, *italic*) correlations with standard errors (in parentheses) between the antibody traits keyhole limpet hemocyanin (KLH)-binding IgM natural antibody titer and KLH-binding IgG natural antibody titer for White and Brown. **Table S15.** Estimates of genetic and phenotypic (*italic*) correlations with standard errors (in parentheses) of the resilience indicators ln(variance), skewness, and autocorrelation based on average batch production for the same life periods with 1-week-intervals and the antibody traits keyhole limpet hemocyanin (KLH)-binding IgM natural antibody titer and KLH-binding IgG natural antibody titer for White. **Table S16.** Estimates of genetic and phenotypic (*italic*) correlations with standard errors (in parentheses) of the resilience indicators ln(variance), skewness, and autocorrelation based on average batch production for the same life periods with 1-week-intervals and the antibody traits keyhole limpet hemocyanin (KLH)-binding IgM natural antibody titer and KLH-binding IgG natural antibody titer for Brown.

## Data Availability

The data that support the findings of this study are not openly available due to commercial sensitivity, and are available from the corresponding authors upon reasonable request after approval of both the authors and Hendrix Genetics.
