# Supplementary material for: The genetics of resilience and its relationships with egg production traits and antibody traits in chickens
Source: Genet Sel Evol. 2024 Mar 19;56:20. doi: 10.1186/s12711-024-00888-5 (PMC10953135; doi:10.1186/s12711-024-00888-5)
Supplement: Supplementary file 1 — Additional file 1: Table S1. Estimates of genetic (below the diagonal) and phenotypic (above the diagonal, italic) correlations with standard errors (in parentheses) between the resilience indicators ln(variance), skewness, and autocorrelation based on average batch production for the same life periods, but with different intervals for White. Table S2. Estimates of genetic (below the diagonal) and phenotypic (above the diagonal, italic) correlations with standard errors (in parentheses) between the resilience indicators ln(variance), skewness, and autocorrelation based on average batch production for the same life periods, but with different intervals for Brown. Table S3. Estimates of genetic (below the diagonal) and phenotypic (above the diagonal, italic) correlations with standard errors (in parentheses) between the resilience indicators ln(variance), skewness, and autocorrelation based on average batch production for 1-week-intervals, but for different life periods for White. Table S4. Estimates of genetic (below the diagonal) and phenotypic (above the diagonal, italic) correlations with standard errors (in parentheses) between the resilience indicators ln(variance), skewness, and autocorrelation based on average batch production for 1-week-intervals, but for different life periods for Brown. Table S5. Estimates of genetic (below the diagonal) and phenotypic (above the diagonal, italic) correlations with standard errors (in parentheses) between the resilience indicators ln(variance), skewness, and autocorrelation based on average batch production for the same life periods with 1-week-intervals for White. Table S6. Estimates of genetic (below the diagonal) and phenotypic (above the diagonal, italic) correlations with standard errors (in parentheses) between the resilience indicators ln(variance), skewness, and autocorrelation based on average batch production for the same life periods with 1-week-intervals for Brown. Table S7. Estimates of genetic and phenotypic ( [file 12711_2024_888_MOESM1_ESM.pdf]

The genetics of resilience and its relationships with egg production traits and antibody traits in chickens

Tom V.L. Berghof, Nicolas Bedere, Katrijn Peeters, Marieke Poppe, Jeroen Visscher, and Han A. Mulder

Additional file 1 Tables S1 to S16

Table S1. Estimates of genetic (below the diagonal) and phenotypic (above the diagonal, italic) correlations with standard errors (in parentheses) between the resilience indicators ln(variance), skewness, and autocorrelation based on average batch production for the same life periods, but with different intervals for White.

				phenotypic correlation		
	**trait**	**period**	**interval**	**1 week**	**2 weeks**	**3 weeks**
**genetic correlations**	**ln(variance)**	**25-83**	**1 week**	-	[a]	*0.93 (0.001)*
			**2 weeks**	[a]	-	[a]
			**3 weeks**	0.97 (0.01)	[a]	-
		**83-end**	**1 week**	-	*0.93 (0.001)*	*0.83 (0.002)*
			**2 weeks**	0.99 (0.003)	-	*0.86 (0.002)*
			**3 weeks**	0.98 (0.01)	0.999 (0.004)	-
	**skewness**	**25-83**	**1 week**	-	*0.82 (0.002)*	*0.70 (0.003)*
			**2 weeks**	0.89 (0.02)	-	*0.82 (0.002)*
			**3 weeks**	0.87 (0.03)	0.97 (0.01)	-
		**83-end**	**1 week**	-	[b]	[b]
			**2 weeks**	[b]	-	[b]
			**3 weeks**	[b]	[b]	-
	**autocorrelation**	**25-83**	**1 week**	-	*0.72 (0.003)*	*0.56 (0.004)*
			**2 weeks**	0.90 (0.03)	-	[a]
			**3 weeks**	0.86 (0.05)	[a]	-
		**83-end**	**1 week**	-	[b]	[b]
			**2 weeks**	[b]	-	[b]
			**3 weeks**	[b]	[b]	-

[a] Analysis did not converge.
[b] Not tested, because one or both traits were not heritable (i.e. not significantly different from zero).

Table S2. Estimates of genetic (below the diagonal) and phenotypic (above the diagonal, italic) correlations with standard errors (in parentheses) between the resilience indicators ln(variance), skewness, and autocorrelation based on average batch production for the same life periods, but with different intervals for Brown.

				phenotypic correlation		
	**trait**	**period**	**interval**	**1 week**	**2 weeks**	**3 weeks**
**genetic correlations**	**ln(variance)**	**25-83**	**1 week**	-	[a]	*0.95 (0.001)*
			**2 weeks**	[a]	-	[a]
			**3 weeks**	0.98 (0.01)	[a]	-
		**83-end**	**1 week**	-	*0.93 (0.001)*	*0.86 (0.004)*
			**2 weeks**	0.99 (0.01)	-	[a]
			**3 weeks**	0.95 (0.07)	[a]	-
	**skewness**	**25-83**	**1 week**	-	*0.87 (0.001)*	*0.76 (0.002)*
			**2 weeks**	0.94 (0.03)	-	[a]
			**3 weeks**	0.90 (0.05)	[a]	-
		**83-end**	**1 week**	-	[b]	[b]
			**2 weeks**	[b]	-	[b]
			**3 weeks**	[b]	[b]	-
	**autocorrelation**	**25-83**	**1 week**	-	*0.76 (0.002)*	*0.57 (0.004)*
			**2 weeks**	0.84 (0.04)	-	*0.79 (0.002)*
			**3 weeks**	0.84 (0.04)	0.68 (0.06)	-
		**83-end**	**1 week**	-	[b]	[b]
			**2 weeks**	[b]	-	[b]
			**3 weeks**	[b]	[b]	-

[a] Analysis did not converge.
[b] Not tested, because one or both traits were not heritable (i.e. not significantly different from zero).

Table S3. Estimates of genetic (below the diagonal) and phenotypic (above the diagonal, italic) correlations with standard errors (in parentheses) between the resilience indicators ln(variance), skewness, and autocorrelation based on average batch production for 1-week-intervals, but for different life periods for White.

			phenotypic correlations	
	**trait**	**period**	**25-83**	**83-end**
**genetic correlations**	**ln(variance)**	**25-83**	-	*0.10 (0.01)*
		**83-end**	0.80 (0.04)	-
	**skewness**	**25-83**	-	[a]
		**83-end**	[a]	-
	**autocorrelation**	**25-83**	-	*0.17 (0.01)*
		**83-end**	0.73 (0.07)	-

[a] Not tested, because one or both traits were not heritable (i.e. not significantly different from zero).

Table S4. Estimates of genetic (below the diagonal) and phenotypic (above the diagonal, italic) correlations with standard errors (in parentheses) between the resilience indicators ln(variance), skewness, and autocorrelation based on average batch production for 1-week-intervals, but for different life periods for Brown.

			phenotypic correlations	
	**trait**	**period**	**25-83**	**83-end**
**genetic correlations**	**ln(variance)**	**25-83**	-	*-0.04 (0.01)*
		**83-end**	0.76 (0.07)	-
	**skewness**	**25-83**	-	*-0.03 (0.01)*
		**83-end**	-0.46 (0.13)	-
	**autocorrelation**	**25-83**	-	*0.19 (0.01)*
		**83-end**	0.80 (0.08)	-

Table S5. Estimates of genetic (below the diagonal) and phenotypic (above the diagonal, italic) correlations with standard errors (in parentheses) between the resilience indicators ln(variance), skewness, and autocorrelation based on average batch production for the same life periods with 1-week-intervals for White.

			phenotypic correlation		
	**trait**	**period**	**ln(variance)**	**skewness**	**autocorrelation**
		**25-end**	-	*-0.32 (0.01)*	*0.56 (0.01)*
	**ln(variance)**	**25-83**	-	*-0.33 (0.01)*	*0.42 (0.01)*
		**83-end**	-	[a]	0.17 (0.01)
		**25-end**	-0.71 (0.05)	-	0.06 (0.01)
**genetic correlation**	**skewness**	**25-83**	-0.46 (0.07)	-	0.03 (0.01)
		**83-end**	[a]	-	[a]
		**25-end**	0.32 (0.06)	0.07 (0.08)	-
	**autocorrelation**	**25-83**	-0.01 (0.08)	-0.21 (0.09)	-
		**83-end**	0.18 (0.08)	[a]	-

[a] Not tested, because one or both traits were not heritable (i.e. not significantly different from zero).

Table S6. Estimates of genetic (below the diagonal) and phenotypic (above the diagonal, italic) correlations with standard errors (in parentheses) between the resilience indicators ln(variance), skewness, and autocorrelation based on average batch production for the same life periods with 1-week-intervals for Brown.

			phenotypic correlation		
	**trait**	**period**	**ln(variance)**	**skewness**	**autocorrelation**
genetic correlation	ln(variance)	25-end	-	*-0.29 (0.01)*	*0.58 (0.004)*
		25-83	-	*-0.32 (0.01)*	*0.51 (0.005)*
		83-end	-	*-0.07 (0.01)*	*0.15 (0.01)*
	skewness	25-end	-0.63 (0.09)	-	*0.06 (0.01)*
		25-83	-0.67 (0.11)	-	*0.04 (0.01)*
		83-end	0.65 (0.09)	-	*0.10 (0.01)*
	autocorrelation	25-end	0.34 (0.08)	-0.32 (0.13)	-
		25-83	0.14 (0.10)	-0.55 (0.15)	-
		83-end	0.42 (0.11)	0.36 (0.13)	-

Table S7. Estimates of genetic and phenotypic (italic) correlations with standard errors (in parentheses) between the resilience indicators ln(variance), skewness, and autocorrelation based on average batch production and expected individual production for the same life periods with 1-week-intervals for White and Brown.

		**genetic correlation**		***phenotypic correlation***	
**trait**	**period**	**White**	**Brown**	**White**	**Brown**
**ln(variance)**	**25-83**	0.95 (0.01)	0.98 (0.01)	*0.88 (0.001)*	*0.90 (0.001)*
	**83-end**	0.996 (0.002)	0.99 (0.01)	*0.92 (0.001)*	*0.81 (0.002)*
**skewness**	**25-83**	0.27 (0.08)	0.001 (0.14)	*0.52 (0.004)*	*0.55 (0.004)*
	**83-end**	[a]	0.99 (0.01)	*[a]*	*0.78 (0.002)*
**autocorrelation**	**25-83**	0.62 (0.06)	0.73 (0.06)	*0.67 (0.003)*	*0.70 (0.003)*
	**83-end**	0.92 (0.02)	0.97 (0.02)	*0.85 (0.001)*	*0.84 (0.002)*

[a] Not tested, because one or both traits were not heritable (i.e. not significantly different from zero).

Table S8. Estimates of variance components (σ^2), heritability (h^2), and maternal environmental effect (m^2) for the full production cycle (i.e. all eggs between start and end), the early production cycle (i.e. between start and 25 weeks of age), the 'traditional' production cycle (i.e. between 25 and 83 weeks of age), and the time period after the 'traditional' production cycle (i.e. between 83 weeks of age and end) with standard errors (SE) for White and Brown.

trait	line	σ_a^2 (SE)	σ_m^2 (SE)	σ_e^2 (SE)	σ_p^2 (SE)	h^2 (SE)	m^2 (SE)
**full**	**White**	405.97 (31.87)	NS	2030.85 (24.33)	2436.80 (23.54)	0.17 (0.01)	NS
	**Brown**	501.34 (57.23)	78.59 (17.66)	3965.68 (45.14)	3965.68 (45.14)	0.11 (0.01)	0.02 (0.004)
**early**	**White**	20.12 (0.91)	NS	23.64 (0.48)	43.75 (0.57)	0.46 (0.02)	NS
	**Brown**	18.62 (1.17)	0.90 (0.21)	32.69 (0.66)	52.21 (0.63)	0.36 (0.02)	0.02 (0.004)
**25-83**	**White**	106.12 (10.99)	5.25 (2.66)	856.90 (9.28)	968.26 (8.76)	0.11 (0.01)	0.01 (0.003)
	**Brown**	211.10 (28.57)	46.91 (10.13)	2437.13 (25.54)	2695.10 (23.08)	0.08 (0.01)	0.02 (0.004)
**83-end**	**White**	99.67 (7.33)	NS[a]	467.27 (5.57)	566.94 (5.47)	0.18 (0.01)	NS[a]
	**Brown**	59.20 (5.63)	5.65 (1.62)	370.85 (4.32)	435.70 (3.95)	0.14 (0.01)	0.01 (0.004)

NS: maternal environmental effect not significantly different from zero.

[a] Tended to be significantly different from zero (i.e. $0.05 < p \leq 0.10$).

Table S9. Estimates of genetic (below the diagonal) and phenotypic (above the diagonal, italic) correlations with standard errors (in parentheses) between egg production traits for the full production cycle (i.e. all eggs between start and end), the early production cycle (i.e. between start and 25 weeks of age), the 'traditional' production cycle (i.e. between 25 and 83 weeks of age), and the time period after the 'traditional' production cycle (i.e. between 83 weeks of age and end) for White.

		phenotypic correlation			
	**trait**	**full**	**early**	**25-83**	**83-end**
**genetic correlation**	**full**	-	*0.25 (0.01)*	*0.91 (0.001)*	*0.82 (0.002)*
	**early**	0.21 (0.04)	-	*0.17 (0.01)*	*0.02 (0.01)*
	**25-83**	0.93 (0.01)	0.03 (0.05)	-	*0.52 (0.005)*
	**83-end**	0.89 (0.01)	-0.06 (0.04)	0.75 (0.03)	-

Table S10. Estimates of genetic (below the diagonal) and phenotypic (above the diagonal, italic) correlations with standard errors (in parentheses) between egg production traits for the full production cycle (i.e. all eggs between start and end), the early production cycle (i.e. between start and 25 weeks of age), the 'traditional' production cycle (i.e. between 25 and 83 weeks of age), and the time period after the 'traditional' production cycle (i.e. between 83 weeks of age and end) for Brown.

		phenotypic correlation			
	**trait**	**full**	**early**	**25-83**	**83-end**
**genetic correlation**	**full**	-	*0.33 (0.01)*	*0.96 (0.0004)*	*0.72 (0.003)*
	**early**	0.28 (0.06)	-	*0.27 (0.01)*	*0.05 (0.01)*
	**25-83**	0.97 (0.005)	0.03 (0.05)	-	*0.53 (0.004)*
	**83-end**	0.89 (0.02)	-0.03 (0.06)	0.82 (0.04)	-

Table S11. Estimates of genetic and phenotypic (italic) correlations with standard errors (in parentheses) between the resilience indicators ln(variance), skewness, and autocorrelation based on average batch production for the same life periods with 1-week-intervals and egg production traits for the full production cycle (i.e. all eggs between start and end), the early production cycle (i.e. between start and 25 weeks of age), the 'traditional' production cycle (i.e. between 25 and 83 weeks of age), and the time period after the 'traditional' production cycle (i.e. between 83 weeks of age and end) for White.

		period	full	early	25-83	83-end
**genetic correlation**	**ln(variance)**	**25-83**	-0.82 (0.02)	0.05 (0.05)	-0.72 (0.04)	-0.88 (0.02)
		**83-end**	-0.62 (0.05)	0.10 (0.05)	-0.43 (0.06)	-0.81 (0.03)
	**skewness**	**25-83**	0.11 (0.08)	-0.15 (0.06)	-0.05 (0.09)	0.33 (0.08)
		**83-end**	[a]	[a]	[a]	[a]
	**autocorrelation**	**25-83**	-0.04 (0.07)	-0.06 (0.06)	0.25 (0.09)	-0.23 (0.07)
		**83-end**	-0.16 (0.08)	-0.07 (0.07)	-0.03 (0.09)	-0.22 (0.08)
**phenotypic correlation**	**ln(variance)**	**25-83**	*-0.60 (0.004)*	*-0.01 (0.01)*	*-0.58 (0.004)*	*-0.48 (0.005)*
		**83-end**	*-0.13 (0.01)*	*0.04 (0.01)*	*0.06 (0.01)*	*-0.34 (0.01)*
	**skewness**	**25-83**	*-0.11 (0.01)*	*-0.05 (0.01)*	*-0.15 (0.01)*	*-0.02 (0.01)*
		**83-end**	*[a]*	*[a]*	*[a]*	*[a]*
	**autocorrelation**	**25-83**	*-0.37 (0.01)*	*-0.04 (0.01)*	*-0.37 (0.01)*	*-0.27 (0.01)*
		**83-end**	*-0.24 (0.01)*	*-0.03 (0.01)*	*-0.15 (0.01)*	*-0.30 (0.01)*

Table S12. Estimates of genetic and phenotypic (italic) correlations with standard errors (in parentheses) between the resilience indicators ln(variance), skewness, and autocorrelation based on average batch production for the same life periods with 1-week-intervals and egg production traits for the full production cycle (i.e. all eggs between start and end), the early production cycle (i.e. between start and 25 weeks of age), the 'traditional' production cycle (i.e. between 25 and 83 weeks of age), and the time period after the 'traditional' production cycle (i.e. between 83 weeks of age and end) for Brown.

		period	full	early	25-83	83-end
**genetic correlation**	**ln(variance)**	**25-83**	-0.84 (0.03)	-0.14 (0.07)	-0.83 (0.04)	-0.77 (0.04)
		**83-end**	-0.46 (0.08)	0.18 (0.07)	-0.42 (0.09)	-0.59 (0.07)
	**skewness**	**25-83**	0.53 (0.14)	-0.21 (0.11)	0.49 (0.15)	0.70 (0.11)
		**83-end**	-0.95 (0.04)	0.06 (0.07)	-0.91 (0.05)	-0.98 (0.02)
	**autocorrelation**	**25-83**	0.22 (0.10)	-0.12 (0.08)	0.38 (0.11)	-0.02 (0.09)
		**83-end**	-0.25 (0.10)	-0.04 (0.09)	-0.11 (0.11)	-0.42 (0.09)
**phenotypic correlation**	**ln(variance)**	**25-83**	*-0.59 (0.004)*	*-0.06 (0.01)*	*-0.59 (0.004)*	*-0.42 (0.01)*
		**83-end**	*0.10 (0.01)*	*0.06 (0.01)*	*0.18 (0.01)*	*-0.10 (0.01)*
	**skewness**	**25-83**	*-0.16 (0.01)*	*-0.11 (0.01)*	*-0.21 (0.01)*	*0.04 (0.01)*
		**83-end**	*-0.35 (0.01)*	*-0.02 (0.01)*	*-0.25 (0.01)*	*-0.50 (0.005)*
	**autocorrelation**	**25-83**	*-0.33 (0.01)*	*-0.06 (0.01)*	*-0.35 (0.01)*	*-0.18 (0.01)*
		**83-end**	*-0.28 (0.01)*	*-0.05 (0.01)*	*-0.21 (0.01)*	*-0.35 (0.01)*

Table S13. Estimates of variance components (σ^2), heritability (h^2), and maternal environmental effect (m^2) of the antibody traits keyhole limpet hemocyanin (KLH)-binding IgM natural antibody titer and KLH-binding IgG natural antibody titer with standard errors (SE) for White and Brown.

trait	line	σ_a^2 (SE)	σ_m^2 (SE)	σ_e^2 (SE)	σ_p^2 (SE)	h^2 (SE)	m^2 (SE)
**IgM**	**White**	0.41 (0.06)	NS	0.83 (0.04)	1.24 (0.04)	0.33 (0.04)	NS
	**Brown**	0.38 (0.06)	NS	0.66 (0.04)	1.04 (0.04)	0.37 (0.05)	NS
**IgG**	**White**	0.11 (0.03)	NS	1.56 (0.04)	1.67 (0.04)	0.07 (0.02)	NS
	**Brown**	0.34 (0.07)	NS	1.27 (0.06)	1.61 (0.06)	0.21 (0.04)	NS

NS: maternal environmental effect not significantly different from zero.

Table S14. Estimates of genetic (below the diagonal) and phenotypic (above the diagonal, italic) correlations with standard errors (in parentheses) between the antibody traits keyhole limpet hemocyanin (KLH)-binding IgM natural antibody titer and KLH-binding IgG natural antibody titer for White and Brown.

		phenotypic correlation			
		**White**		**Brown**	
	**trait**	**IgM**	**IgG**	**IgM**	**IgG**
**genetic correlation**	**IgM**	-	*0.26 (0.02)*	-	*0.24 (0.02)*
	**IgG**	0.84 (0.07)	-	0.24 (0.12)	-

Table S15. Estimates of genetic and phenotypic (italic) correlations with standard errors (in parentheses) of the resilience indicators ln(variance), skewness, and autocorrelation based on average batch production for the same life periods with 1-week-intervals and the antibody traits keyhole limpet hemocyanin (KLH)-binding IgM natural antibody titer and KLH-binding IgG natural antibody titer for White.

		**genetic correlation**		***phenotypic correlation***	
	**period**	**IgM**	**IgG**	**IgM**	**IgG**
**ln(variance)**	**25-83**	-0.09 (0.11)	0.06 (0.17)	*0.01 (0.02)*	*-0.01 (0.02)*
	**83-end**	-0.12 (0.12)	-0.02 (0.18)	*0.03 (0.02)*	*0.01 (0.02)*
**skewness**	**25-83**	0.08 (0.13)	-0.18 (0.19)	*0.004 (0.02)*	*0.0002 (0.02)*
	**83-end**	[a]	[a]	[a]	[a]
**autocorrelation**	**25-83**	0.16 (0.12)	0.21 (0.18)	*-0.02 (0.02)*	*-0.03 (0.02)*
	**83-end**	0.15 (0.15)	0.13 (0.21)	*-0.001 (0.02)*	*-0.01 (0.02)*

[a] Not tested, because the resilience indicator trait was not heritable (i.e. not significantly different from zero).

Table S16. Estimates of genetic and phenotypic (italic) correlations with standard errors (in parentheses) of the resilience indicators ln(variance), skewness, and autocorrelation based on average batch production for the same life periods with 1-week-intervals and the antibody traits keyhole limpet hemocyanin (KLH)-binding IgM natural antibody titer and KLH-binding IgG natural antibody titer for Brown.

		**genetic correlation**		***phenotypic correlation***	
	**period**	**IgM**	**IgG**	**IgM**	**IgG**
**ln(variance)**	**25-83**	-0.03 (0.30)	-0.27 (0.32)	*[a]*	*[a]*
	**83-end**	-0.21 (0.34)	-0.24 (0.39)	*[a]*	*[a]*
**skewness**	**25-83**	-0.16 (0.42)	0.16 (0.48)	*[a]*	*[a]*
	**83-end**	0.28 (0.34)	-0.09 (0.39)	*[a]*	*[a]*
**autocorrelation**	**25-83**	-0.09 (0.31)	-0.15 (0.33)	*[a]*	*[a]*
	**83-end**	0.03 (0.42)	0.56 (0.42)	*[a]*	*[a]*

[a] Phenotypic correlation could not be estimated, because traits were collected on different individuals.
